# Preclinical development of T-cell receptor-engineered T-cell therapy targeting the 5T4 tumor antigen on renal cell carcinoma

**DOI:** 10.1007/s00262-019-02419-4

**Published:** 2019-11-04

**Authors:** Yuexin Xu, Alicia J. Morales, Michael J. Cargill, Andrea M. H. Towlerton, David G. Coffey, Edus H. Warren, Scott S. Tykodi

**Affiliations:** 1 Clinical Research Division, Fred Hutchinson Cancer Research Center, Seattle, WA USA; 2 Department of Pathology, University of Washington School of Medicine, Seattle, WA USA; 3 Division of Medical Oncology, Department of Medicine, University of Washington, Seattle, WA USA

**Keywords:** 5T4, Trophoblast glycoprotein, Renal cell carcinoma, T-cell receptor, Adoptive cell therapy, Antigen processing

## Abstract

5T4 (trophoblast glycoprotein, TPBG) is a transmembrane tumor antigen expressed on more than 90% of primary renal cell carcinomas (RCC) and a wide range of human carcinomas but not on most somatic adult tissues. The favorable expression pattern has encouraged the development and clinical testing of 5T4-targeted antibody and vaccine therapies. 5T4 also represents a compelling and unexplored target for T-cell receptor (TCR)-engineered T-cell therapy. Our group has previously isolated high-avidity CD8^+^ T-cell clones specific for an HLA-A2-restricted 5T4 epitope (residues 17–25; 5T4_p17_). In this report, targeted single-cell RNA sequencing was performed on 5T4_p17_-specific T-cell clones to sequence the highly variable complementarity-determining region 3 (*CDR3*) of T-cell receptor α chain (*TRA*) and β chain (*TRB*) genes. Full-length *TRA* and *TRB* sequences were cloned into lentiviral vectors and transduced into CD8^+^ T-cells from healthy donors. Redirected effector T-cell function against 5T4_p17_ was measured by cytotoxicity and cytokine release assays. Seven unique *TRA*-*TRB* pairs were identified. All seven TCRs exhibited high expression on CD8^+^ T-cells with transduction efficiencies from 59 to 89%. TCR-transduced CD8^+^ T-cells demonstrated redirected cytotoxicity and cytokine release in response to 5T4_p17_ on target-cells and killed 5T4^+^/HLA-A2^+^ kidney-, breast-, and colorectal-tumor cell lines as well as primary RCC tumor cells in vitro. TCR-transduced CD8^+^ T-cells also detected presentation of 5T4_p17_ in *TAP1/2*-deficient T2 target-cells. TCR-transduced T-cells redirected to recognize the 5T4_p17_ epitope from a broadly shared tumor antigen are of interest for future testing as a cellular immunotherapy strategy for HLA-A2^+^ subjects with 5T4^+^ tumors.

**Electronic supplementary material:**

The online version of this article (10.1007/s00262-019-02419-4) contains supplementary material, which is available to authorized users.

## Introduction

Renal cell carcinoma (RCC) accounts for 90% of malignant neoplasms arising in the kidney in adults and is the eighth most common cancer in the United States, with an estimated 73,820 new cases in 2019 [1]. Despite the development of targeted molecular therapies, including tyrosine kinase inhibitors (TKIs) and mammalian target of rapamycin (mTOR) inhibitors, metastatic RCC is generally considered an incurable disease with a 5-year survival rate of only 12% [1]. However, metastatic RCC can be uniquely sensitive to systemic immunotherapy. Both high-dose interleukin-2 (IL-2) [2] and, more recently, combination immune checkpoint blockade with antibodies targeting programmed cell death-protein 1 (PD1) and cytotoxic T-lymphocyte associated protein 4 (CTLA4) [3] have been associated with complete radiographic responses in 5–9% of RCC patients. The anti-tumor effects produced by these agents are thought to be mediated by tumor-reactive T-cell responses.

While the antigens associated with immunotherapy mediated regression of RCC are not well defined, previous studies have identified 5T4 (trophoblast glycoprotein, TPBG) as an RCC-associated antigen of therapeutic interest. 5T4 is highly expressed by placental trophoblasts and a wide range of human carcinomas, including renal, prostate, pancreatic, ovarian, breast, cervical, gastric, and non-small cell lung cancer [4, 5]. Greater than 90% of RCC tumors over-express 5T4 and the expression is maintained on metastatic lesions [6]. 5T4 expression on tumors has been associated with a stem-cell phenotype subpopulation in human non-small cell lung cancers, nasopharyngeal carcinoma [7–9] and with epithelial–mesenchymal transition that may be related to cancer cell motility and metastatic spread [4]. 5T4 protein is undetectable or expressed at a very low level by healthy adult tissues [5, 6, 10].

The favorable expression pattern of 5T4 has encouraged the development and clinical testing of 5T4-targeted antibody–drug conjugates (ADCs) engineered with superantigen [11] or chemotherapy payloads [12] and a recombinant modified vaccinia virus Ankara (MVA) expressing the full-length *5T4* gene (MVA-5T4). MVA-5T4 is the most extensively studied 5T4-target therapy and has been applied to > 580 subjects with colorectal, prostate, and renal cancer [4]. Early phase clinical testing demonstrated MVA-5T4 was able to elicit 5T4-specific serological and T-cell responses in vaccinated cancer subjects [13]. 5T4-targeting by ADC or MVA-5T4 vaccine has not been associated with off-tumor on-target toxicities affecting healthy tissues. However, despite encouraging early phase data, none of these agents have gained regulatory approval as a cancer therapy.

Engineering T-cells to express foreign TCRs or chimeric antigen receptors (CARs) targeting tumor-associated antigens represents a therapy platform with the potential to massively expand tumor-reactive T-cells in cancer subjects. The recent clinical success of engineered T-cells expressing CARs specific for CD19 achieving complete remissions of refractory acute lymphocytic leukemia [14] and non-Hodgkin lymphoma [15] has created intense interest to extend engineered T-cells as a therapeutic modality to solid tumor targets. TCR-engineered T-cell therapy targeting the cancer/testis antigen NY-ESO-1 in melanoma and synovial sarcoma [16, 17], and more recently TCR engineered T-cells targeting human papillomavirus (HPV) antigens E6 or E7 in HPV^+^ cancers [18, 19] associated with partial tumor responses in some patients establish proof-of-concept for the therapeutic use of TCR engineered T-cells targeting a single tumor antigen to result in significant tumor regression.

5T4 represents a compelling and unexplored target for TCR-engineered T-cell therapy. Our group has previously isolated high-avidity CD8^+^ T-cell clones from both healthy and kidney cancer donors specific for an HLA-A2-restricted 5T4 epitope (residues 17–25; 5T4_p17_) [10]. In this study, we sequenced the CDR3s from the *TRA* and *TRB* genes isolated from these high-avidity 5T4_p17_-specific clones to identify unique TCRs recognizing 5T4_p17_. We have assessed 5T4_p17_-specific TCR-transduced T-cells from healthy donors for redirected recognition of 5T4_p17_ on target cells, including HLA-A2^+^ human tumor-cell lines and short-term in vitro cultures of primary RCC tumors expressing the 5T4 antigen.

## Materials and methods

### CDR3 domain sequencing for *TRA* and *TRB* genes from 5T4_p17_-specific CD8^+^ T-cell clones

Genomic DNA was isolated using the QIAamp DNA Blood Mini Kit (Qiagen, Hilden, Germany) from 19 CD8^+^ T-cell clones specific for 5T4_p17_ presented by HLA-A2. High throughput-bulk sequencing of the T-cell receptor β chain was performed using the hsTCRB ImmunoSeq kit (Adaptive Biotechnologies, Seattle, WA) at survey level resolution [20] on the Illumina MiSeq platform (v3 150 cycle) in the Genomics Core Facility at the Fred Hutchinson Cancer Research Center. Repertoire analyses were conducted using the LymphoSeq R package (created by D. G. Coffey; http://bioconductor.org/packages/LymphoSeq).

Targeted single-cell *TRA* and *TRB* sequencing were conducted according to methods previously reported [21]. For each clone, 8 or 16 single CD8^+^CD3^+^DAPI^−^ cells were sorted into a 96-well PCR plate. Targeted-reverse transcription of CDR3-regions was conducted on the mRNA transcripts of *TRA* and *TRB* using the One-step RT PCR kit (Qiagen, Hilden, Germany). The cDNA library was PCR-amplified, barcoded [21], pooled and purified with Agencourt AMPure XP beads (Beckman Coulter, Brea, CA). Sequencing was performed for pair-end 250 bp (MiSeq reagent kit v2, 500-cycles, Illumina, San Diego, CA). FASTQ files were de-multiplexed, and CDR3 regions with associated V(D)J region-information were extracted with the MiXCR package [22]. Net charges of CDRregions were computed by the R package “Peptides” [23].

### Cloning full-length *TRA* and *TRB* sequences

Reference V- and C-gene open-reading-frames of *TRA* and *TRB* were obtained from the International Immunogenetics Information System (IMGT) [24, 25]. Codon optimized Vα and Vβ DNA fragments with corresponding CDR3 sequences were then synthesized by the GeneArt Strings DNA Fragments service (Invitrogen, Carlsbad, CA). Each DNA fragment included the following Gibson overhang sequences attached to both ends: Vα 5′: AGGAGACGTGGAAGAAAACCCCGGTCCC; Vα 3′: ACATCCAGAACCCCGACCCTGCAGTGTACCAGCTGCGGGAC; Vβ 5′: TCCCCGAGCTCAATAAAAGAGCCCACAACCCCTCACTCGGCGCGCCGGCCACC; Vβ 3′: GTGTTCCCCCCAGAGGTGGCCGTGTTCGAG. The stop codon of constant region of TCR-β gene (*TRBC*) was deleted and the self-cleaving porcine teschovirus-1 2A sequence.

(P2A: GGTTCCGGAGCCACGAACTTCTCTCTGTTAAAGCAAGCAGGAGACGTGGAAGAAAACCCCGGTCCC) was incorporated between *TRA* and *TRB*, to ensure equal expression of α and β chains. Full-length TCRs were assembled using the Gibson Assembly^®^ Ultra kit (SGI-DNA, La Jolla, CA). TCRs were then cloned into lentiviral vectors pRRLSIN, with the murine stem cell virus (MSCV) promoter to drive expression in human primary T-cells (pRRLSIN, pRSV-REV, pMD2-G, and pMDLg/pRRE were generous gifts of Dr. Philip Greenberg, Seattle, WA) [26]. Constructs were transformed into One Shot^®^ TOP10 Chemically Competent *E. coli* (Invitrogen). Plasmid DNA was extracted using the Endotoxin-free Mini- and Midi-Prep DNA isolation kits (Qiagen).

### Lentiviral packaging and T-cell transduction

Lenti-X 293T virus packaging cells (Clontech Laboratories, Mountain View, CA) were seeded at 60% confluency in RPMI-HEPES supplemented with 10% fetal bovine serum, 2 mmol/L L-glutamine, and 1% penicillin/streptomycin (termed LCL medium). 5T4_p17_-specific TCR encoding lentivirus vectors were co-transfected with packaging plasmids (pRRSIN-TCR: 1.5 μg, pRSV-REV: 1 μg, pMD2-G: 0.5 μg, and pMDLg/pRRE 1 μg) using the Effectene transfection reagent (Qiagen). Media was changed the next day; from day 2 post-transfection, and lentivirus containing supernatants were harvested each day for 2 days and passed through 0.45 μm filters. Viral supernatants were concentrated using Lenti-X viral concentrator (Clontech Laboratories), aliquoted and applied to T-cells for transduction or stored at − 80 °C.

CD8^+^ T-cells were isolated from healthy donor PBMCs using a human CD8^+^ T-cell isolation kit (Miltenyi Biotec, Bergisch Gladbach, Germany) and activated for 4 h with CD3/CD28 Dynabeads^®^ human T-cell expander (Thermo Fisher Scientific, Waltham, MA). Viral supernatant was applied with 6 μg/mL polybrene (Sigma-Aldrich, St. Louis, MO) to T-cells and centrifuged at 1455 × *g* for 90 min at 35 °C. Transduction was repeated the following day. T-cell cultures were maintained in cytotoxic T-lymphocyte culturing media [27] with 50 U/mL recombinant human IL-2 (PeproTech, Rocky Hill, NJ) for 7 days.

### Endogenous *TRA* knockout

Healthy donor CD8^+^ T-cells were stimulated for 2 days with CD3/CD28 Dynabeads^®^ human T-cell expander (Thermo Fisher Scientific). crRNA–tracrRNA duplex was prepared the morning of electroporation. Constant region of *TRA* (TRAC) targeting crRNA (Alt-R^®^ CRISPR-Cas9 crRNA: AGAGTCTCTCAGCTGGTACA [28], Integrated DNA technology, Inc., Coralville, IA) and tracrRNA (Alt-R^®^ CRISPR-Cas9 tracrRNA, Integrated DNA technology, Inc.) were reconstituted to 200 µM with Nuclease-Free Duplex Buffer (Integrated DNA technology, Inc.) and mixed at equimolar concentrations. Oligos were then annealed by heating at 95 °C for 5 min in PCR thermocycler and slowly cooled to room temperature. Alternatively, control crRNA (Alt-R^®^ CRISPR-Cas9 Control Kit, human, Integrated DNA technology, Inc.) was mixed with the tracrRNA the same manner. Duplexes and Cas9 Nuclease V3 (Integrated DNA technology, Inc.) were gently mixed and incubated at room temperature for at least 10 min. 1 million T-cells were resuspended in 20 µL primary-cell nucleofection solution (P3 Primary Cell Nucleofector™ Solution, Lonza, Basel, Switzerland). T-cells were added with Alt-R Cas9 Electroporation Enhancer (Integrated DNA technology, Inc.) to 4 μM, and incubated with 5 µL duplex-Cas9 nuclease mix for 2 min. T-cells were electroporated using a 4D nucleofector (Lonza) with EH115 program. After nucleofection, T-cells were cultured at 10^6^ cells per well in 200 µL prewarmed complete T-cell media. 6 h post nucleofection, T-cells were transduced with the corresponding lentivirus as described in the previous section.

### Flow cytometry and T-cell expansion

To assess 5T4_p17_-specific TCR expression, lentiviral-transduced T-cell cultures were stained by DAPI, 1:20 dilution of APC-Cy7-labeled anti-CD3 mAb, (clone SK7; BD Biosciences, San Jose, CA), 1:20 dilution of FITC-labeled anti-CD8 mAb (clone RPA-T8; BD Biosciences) as well as 10 μg/mL of APC-labeled 5T4_p17_/HLA-A2 tetramer (TET, generated by the Immune Monitoring Core Laboratory at our center) and analyzed by flow cytometry (BD FACSymphony™, BD biosciences, San Jose, CA). Viable TET^+^CD8^+^CD3^+^ T-cells were flow-sorted (BD FACSAria™ II, BD biosciences) and expanded as described [29] in cytotoxic T-lymphocyte culturing media supplemented with 50 U/mL IL-2 (PeproTech) and 5 ng/mL Interleukin-15 (PeproTech).

### Cytotoxicity and ELISA assays

For some experiments, T2- and LCL-targets were infected with wild-type MVA or MVA-5T4 (Oxford Biomedica, Oxford, UK) at a 10:1 multiplicity of infection in serum-free RPMI media at 37 °C for 1 h. Media were then adjusted to 10% serum. To confirm 5T4 expression on surface after MVA-5T4 infection, target cells were stained with a 5T4-specific mAb (clone 524744; R&D systems, Minneapolis, MN) at 1 μg/mL followed by a 1:50 dilution of a secondary PE-labeled anti-mouse IgG1 mAb (clone A85-1; BD Biosciences) for analysis by flow cytometry. T-cell and target-cell co-culture supernatants were harvested after 4 h to perform chromium-release assays as previously described [30]. Supernatants after 16 h of T-cell target-cell co-culture were assayed for interferon-γ (IFN-γ) or tumor necrosis factor-α (TNF-α) by enzyme-linked immunosorbent assays (ELISA) according to the manufacturer’s protocol (BosterBio, Pleasanton, CA).

### HLA-A2 stabilization assay

Synthetic peptide 5T4_p17_ RLARLALVL, and alanine-substituted variant sequences ALARLALVL (R1A), RLAALALVL (R4A), RLARAALVL (L5A), RLARLAAVL (L7A), RLARLALAL (V8A) at a purity > 90% (Genscript, Piscataway, NJ) were dissolved in 100% dimethyl sulfoxide (Invitrogen) and stored at 4 °C. Aliquots of 2 × 10^5^ T2 cells were cultured in serum-free RPMI media supplemented with human β_2_-microglobulin (BosterBio) at 5 μg/mL and the test peptide. After overnight incubation at 37 °C, cells were stained with an APC-labeled HLA-A2-specific mAb (clone BB7.2, BD Biosciences) at dilution of 1:50 with DAPI and analyzed by flow cytometry.

## Results

### Sequencing TCRs from 5T4_p17_-specific CD8^+^ T-cell clones reveals similarity in CDR3 region and V(D)J gene usage

Our group has previously characterized high-avidity 5T4_p17_-specific CD8^+^ T-cell clones isolated from seven T-cell lines stimulated in vitro with varying concentrations of 5T4_p17_. These lines were derived from leukapheresis products of four HLA-A2^+^ donors (three healthy donors, HD_A, HD_B, and HD_C; and one donor with 5T4^+^ metastatic kidney cancer, KCD_D, 10^7^ responder T-cells for each condition) [10]. To assess the TCR-β gene usage, we sequenced CDR3 regions from the *TCR*-*β* gene (*TRB*-*CDR3*) expressed by 19 5T4_p17_-specific CD8^+^ T-cell clones. Two clones had two *TRB*-*CDR3* sequences—HD_A, clone #1 and HD_B, clone #20. In each case, one *TRB*-*CDR3* sequence was identical to the other clones isolated from the same T-cell line (Table 1). Our hypothesis to account for two *TRB*-*CDR3* sequences in these two “clones” was flow sorting of doublets representing a 5T4_p17_-specific CD8^+^ T-cell that stained tetramer-positive adherent to an unrelated passenger T-cell. All three clones isolated from the HD_C/0.1 μg/mL-stimulated T-cells had a nonproductive *TRB* rearrangement (“null” allele) and a shared in-frame *TRB*-*CDR3* sequence (CASSYMGPEAFF; Table 1).

**Table 1:** High throughput bulk sequencing of the T-cell receptor β chain for 5T4_p17_-specific CD8^+^ T-cell clones isolated from healthy or kidney cancer donors

Donor	[5T4_p17_] stimulation (µg/mL)	Clone	*TRB*-CDR3 sequence	Frequency (%)	*TRA/B* single-cell sequencing
HD_A	10	1	**CASSELPAGGTNEQFF**	62.70	−
			CASSIYGETGTEAFF	35.20	
		2	**CASSELPAGGTNEQFF**	97.94	+
	0.1	7	**CASSFFSNTGELFF**	99.20	−
		8	**CASSFFSNTGELFF**	98.17	−
		12	**CASSFFSNTGELFF**	98.52	−
		15	**CASSFFSNTGELFF**	97.94	+
HD_B	10	17	**CASSQVSGYEQYF**	95.48	−
		19	**CASSQVSGYEQYF**	99.54	+
	0.1	20	CASSLTSQGFQPQHF	65.25	+
			**CASSPQGDNEQFF**	34.06	
		21	**CASSPQGDNEQFF**	99.02	+
		24	**CASSPQGDNEQFF**	95.96	−
HD_C	10	1	**CASMDLAFKQYF**	97.50	−
		3	**CASMDLAFKQYF**	98.41	+
		6	**CASMDLAFKQYF**	98.41	−
		11	**CASMDLAFKQYF**	98.74	−
	0.1	17	Null	63.66	+
			**CASSYMGPEAFF**	34.79	
		18	Null	62.21	−
			**CASSYMGPEAFF**	36.81	
		20	Null	62.80	−
			**CASSYMGPEAFF**	36.48	
KCD_D	10	6	**CASSFLTDTQYF**	96.75	+

The bolded sequences were shared among clones from the same donor and peptide stimulation concentration

*TRB* T-cell receptor beta, *CDR3* complementarity-determining region 3, *TRA* T-cell receptor alpha, *HD* healthy donor, *KCD* kidney cancer donor

Based on this interpretation, our analysis of 19 5T4_p17_-specific CD8^+^ T-cell clones identified seven unique *TRB*-CDR3 sequences, with only one unique sequence corresponding to each of the seven originating T-cell lines. This result indicates the precursor frequency for each T-cell line with unique *TRB*-CDR3 could be as low as one cell out of the initial input 10^7^ CD8^+^ T-cells. To further infer precursor frequency, we sequenced *TRB*-CDR3 from flow-sorted CD8^+^ T-cells purified from available donor leukapheresis products (HD_A, HD_B, and KCD_D) as well as two additional PBMC samples and a tumor-infiltrating lymphocyte sample also from donor KCD_D (Supplementary Table 1 and Supplementary Fig. 1). KCD_D was a 60-year-old male subject with metastatic clear-cell RCC, treated with high-dose IL-2 and multiple lines of targeted drug. Our analyses did not detect the corresponding *TRB*-*CDR3* sequences associated with 5T4_p17_-specificity from any of the samples collected from donors HD_A, HD_B, or KCD_D. These data suggest an upper boundary for precursor frequencies for 5T4_p17_-specific clones at < 1 in 1–1.5 × 10^5^ CD8^+^ T-cells.

To identify the *TCR*-*α* gene CDR3 region (*TRA*-CDR3) pairing with each of the unique *TRB*-CDR3, targeted single-cell RNA-Seq of the CDR3 regions of *TRA* and *TRB* genes was then performed. We discovered seven unique single *TRA*-CDR3 sequences that each paired with one of the seven *TRB*-CDR3s (Supplementary Table 2). Single-cell RNA-Seq analysis of HD_C, clone #17 confirmed both the nonproductive- and productive-rearrangement of *TRB* within each cell. Single-cell RNA-Seq analysis of HD_B, clone 20 resolved two separate cell populations, each expressing one of the identified *TRB*-CDR3 sequences consistent with our hypothesis of a flow-sorted doublet.

**Fig. 1:**
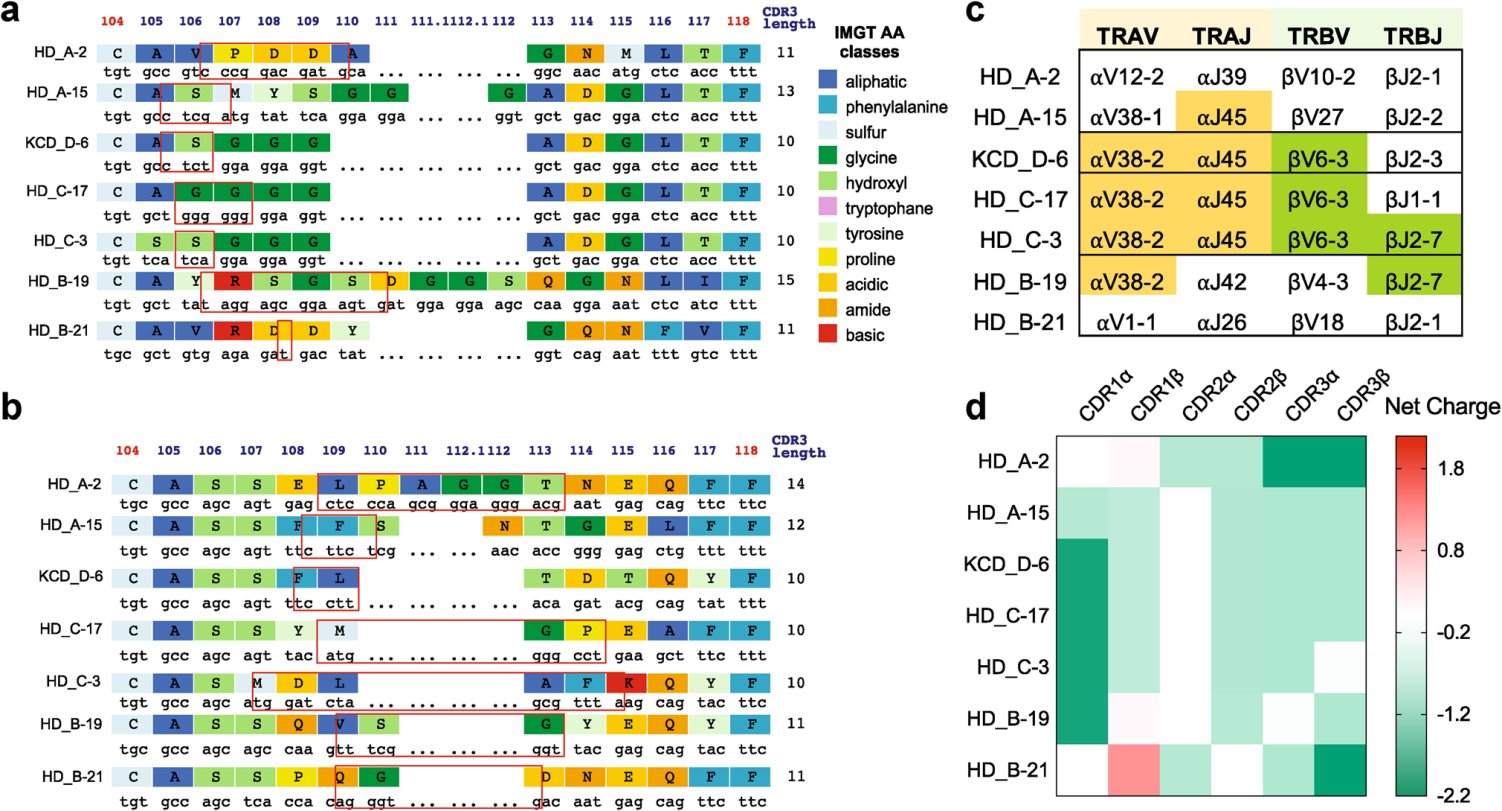
Characteristics of 5T4_p17_ -specific CDR3 regions of *TRA* and *TRB*. IMGT junction analysis is shown for **a***TRA*-CDR3 regions and **b***TRB*-CDR3 regions. Red boxes indicate N nucleotides and/or D regions (*TRB*) between V gene and J gene encoded sequence. **c** V- and J-gene usage for *TRA* and *TRB* are shown. Color shading identifies common gene use between TCRs. **d** The net charge for CDR1, 2, and 3 amino acid sequences from 5T4_17–25_-specific TCRs are shown

Our analysis did not reveal public TCR sequences shared between donors. However, we did observe CDR3 sequences with a high degree of homology. The *TRA*-CDR3 region of KCD_D-6 and HD_C-17 differed by only one amino acid at position 106 (serine of KCD_D-6, glycine of HD_C-17), while KCD_D-6 and HD_C-3 differed only at position 105 (alanine of KCD_D-6, serine of HD_C-3, Fig. 1a). V(D)J gene analysis with the IMGT V-quest [31, 32] also revealed common gene-segment usage among subsets of 5T4_p17_-specific TCRs. *TRAV38*-*2* and *TRAJ45* were both shared by 4 of 7 TCRs, and *TRBV6*-*3* and *TRBJ2*-*7* were shared by 3 and 2 of 7 TCRs, respectively (Fig. 1c).

TCRs use a conserved mechanism to bind to HLA-A2 despite lack of consensus motifs in CDR1/CDR2 regions. Two unique positive-charged residues (R65 and K66) on the HLA-A2 α1-helix are crucial elements to interact with the negatively charged residues (Asp and Glu) in CDR1/CDR2 domains of HLA-A2-specific TCRs [33]. Therefore, V(D)J gene-segment usage for HLA-A2 specific tumor antigen recognition is biased [34]. Although the most frequently used V gene-segments in 5T4_p17_-specific clones (*TRAV38* and *TRBV6*-*3*) are not among the most common HLA-A2 biased *V* genes, the CDR1 amino acid sequences of *TRAV38* and *TRBV6*-*3* are negatively charged at − 2 and − 0.9, respectively, consistent with preferential binding with HLA-A2 (Supplementary Table 2 and Fig. 1d). The amino acid sequences of 5T4_p17_-specific CDR3 regions are also negatively charged (Fig. 1d), except for CDR3α on HD_B-19 and CDR3β on HD_C-3, indicating a direct interaction of CDR3s and the positively charged 5T4_p17_ epitope (RLARLALVL, with two positively charged arginine residues).

## 5T4_p17_-specific TCRs exhibit robust and stable expression on CD8^+^ T-cells from healthy donors

**Fig. 2:**
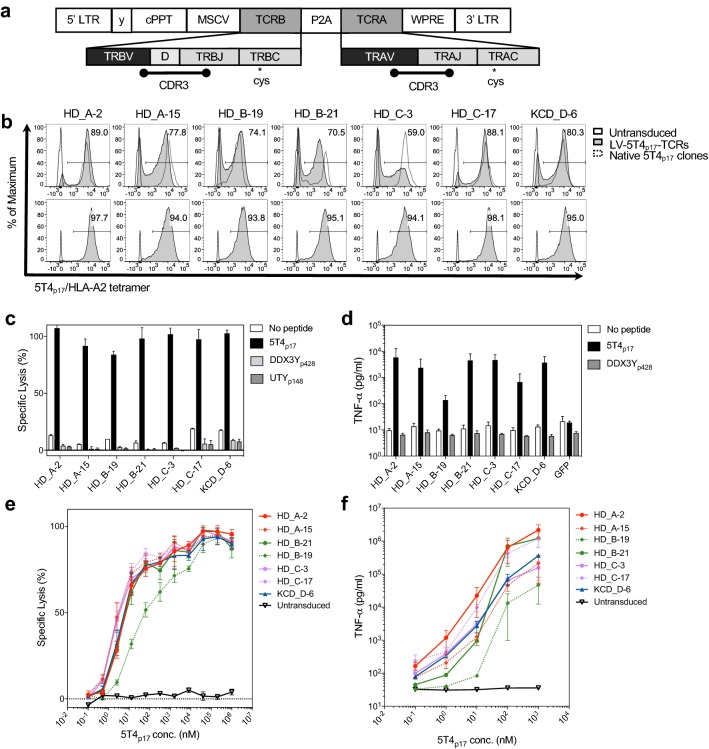
5T4_p17_-specific TCRs expression on CD8^+^ T-cells from healthy donors and redirected effector functions against peptide-pulsed T2 targets. **a** Vector structure of 5T4_p17_-specific *TCR* assembly: *TRB* and *TRA* are transcribed under control of the MSCV promotor; P2A cleaves the primary transcripts to equal molar quantity of *TRA* and *TRB* in the cytosol. The enlarged graph indicates the gene segments and CDR3s, contributing to *TRA* and *TRB* sequence assembly. The relative location for the introduced transmembrane cysteines in *TRAC* and *TRBC* are indicated. **b** Cell surface expression of 5T4_p17_-specific TCRs stained by 5T4_p17_/HLA-A2 tetramer at day 7 post-transduction is shown in comparison to tetramer staining of the native T-cell clone expressing the corresponding TCR (upper panels). Cell surface expression of 5T4_p17_-specific TCRs on healthy donor T-cells is shown after sorting for 5T4_p17_/HLA-A2 tetramer^+^ cells and 12 days expansion (lower panels). CD8^+^ T-cells expressing 5T4_p17_-specific TCRs were tested for recognition of **c** T2 cells pulsed with 10 nM of 5T4_p17_ or control HLA-A2-binding peptides DDX3Y_428–436_ or UTY_148–156_ in a 4-h cytotoxicity assay. The effector: target ratio (E:T) was 10:1. **d** TNF-α release was measured by ELISA in culture supernatants harvested after 18 h co-culture of effector T-cells with T2 cells pulsed with 10 nM of 5T4_p17_ or the control peptide DDX3Y_428–436_ at 10:1 E:T. **e** Effector T-cells were tested for recognition of T2 cells pulsed with 5T4p17 peptide in a 4-h cytotoxicity assay at 10:1 E:T. **f** TNF-α release was measured by ELISA in culture supernatants harvested after 18 h co-culture of effector T-cells with T2 cells pulsed with 5T4_p17_ peptide at 10:1 E:T

We assembled the full sequences of *TRA* and *TRB* genes by adding reference V gene- and C gene-segments to the CDR3s (Supplementary Table 3; Supplementary Table 4). Codon optimized full-length *TCRα* and *β* sequences were designed to incorporate cysteine point mutations in *TRAC* and *TRBC*, respectively (Supplementary Table 5), to crosslink lentiviral encoded 5T4_p17_-specific α and β chains and minimize mispairing with endogenous TCR counterparts (Fig. 2a) [35]. A P2A autocleavage site linked the full-length TRA and TRB genes with the orientation of *TRB* followed by *TRA* [36] to ensure equimolar production. CD8^+^ T-cells from peripheral blood of HLA-A2^+^ healthy donors were isolated and transduced with lentivirus encoding 5T4_p17_-specific TCRs. At day 7 post-transduction, 5T4_p17_-specific TCRs were detected by tetramer staining on 59–89% of transduced T-cells (Fig. 2b). No significant differences were observed in the transduction efficiency from three healthy donors (Supplementary Fig. 2). The mean fluorescent intensity of 5T4_p17_-specific TCR expression on flow-sorted tetramer^+^ cells following 12 days expansion was maintained at levels similar to the primary transduced cells (Fig. 2b).

### CD8^+^ T-cells expressing lentiviral-transduced TCRs exhibit redirected effector functions for 5T4_p17_/HLA-A2

TCR transduced T-cells exhibited potent cytolytic activity for T2 targets pulsed with 10 nM 5T4_p17_ peptide without cross-reactivity for control HLA-A2 binding peptides (DDX3Y_428–436_ FLLDILGAT and UTY_148–156_ KAFQDVLYV) (Fig. 2c) [37]. When tested for recognition of limiting dilutions of 5T4_p17_, six of the 7 TCR-transduced T-cell lines exhibited similar half-maximum lysis of 5T4_p17_ peptide-pulsed T2 targets at concentrations of 1–10 nM peptide (Fig. 2e). After overnight co-culture with T2 target-cells pulsed with 10 nM 5T4_p17_, TCR transduced CD8^+^ T-cells released robust amounts of TNF-α (Fig. 2d) again without cross-reactivity to control HLA-A2 binding peptides. The half-maximum release of TNF-α was observed with ~ 10 nM concentration of 5T4_p17_ peptide for six of the 7 TCRs (Fig. 2f). Five of the seven TCR-transduced T-cell lines produced detectable TNF-α release above background at a 5T4_p17_ peptide concentration as low as 0.1 nM. Comparing with other TCRs, the HD_B-19 TCR-transduced T-cell lines required approximately tenfold higher peptide concentration for equivalent half-maximum lysis and TNF-α release. Similar observations were also made with IFN-γ release (Supplementary Fig. 3).

**Fig. 3:**
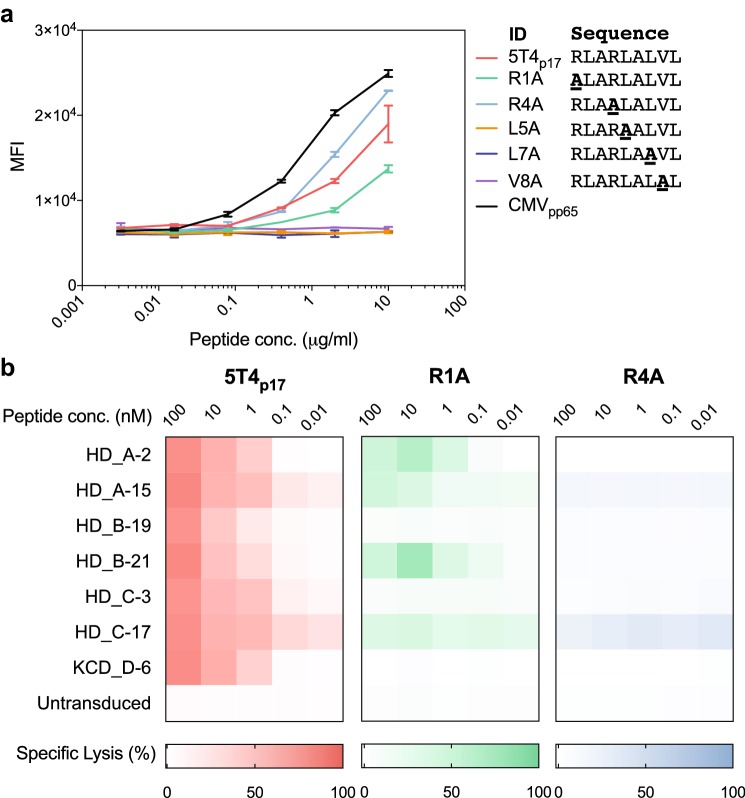
Cytotoxicity of 5T4_p17_-specific TCR transduced CD8^+^ T-cells against T2 pulsed with alanine-substituted 5T4_p17_ peptides. **a** Binding of alanine-substituted 5T4_p17_ peptides to HLA-A2 was determined by stabilization of HLA-A2 on the surface of peptide-pulsed T2 cells. CMV_pp65_ is an HLA-A2^+^ T-cell epitope from CMV and serves as a positive control. Cell surface HLA-A2 was assessed by immunostaining and flow cytometry. **b** CD8^+^ T-cells expressing 5T4_p17_-specific TCRs were tested for recognition of T2 cells pulsed with the progenitor 5T4_p17_ peptide or HLA-A2 binding alanine-substituted variants in a 4-h cytotoxicity assay with a 10:1 E:T

To closely examine the TCR-peptide/major histocompatibility complex (MHC) binding specificity for 5T4_p17_-specific TCRs, we synthesized five 5T4_p17_ peptide variants containing non-alanine residues substituted each to the nonpolar, aliphatic alanine residue, except for the two conserved anchor residues essential for HLA-A2 binding (RLARLALVL; lysine at position 2 and position 9) [38]. MHC binding affinities of alanine-substituted and proband 5T4_p17_ peptides were measured by cell surface HLA-A2 stabilization on T2 cells using flow cytometry (Fig. 3a). The 5T4_p17_, R1A, and R4A peptides stabilized HLA-A2 on the surface of T2 cells with a measurable increase of HLA-A2 staining. The alanine-substituted peptides L5A, L7A, and V8A showed no capacity to bind to HLA-A2 suggesting side chains at these positions may contribute to the peptide–MHC binding interaction.

We then performed a cytotoxicity assay with the seven 5T4_p17_-specific TCR expressing T-cell lines and T2 targets pulsed with the 5T4_p17_, R1A, or R4A peptides. Switching the positively charged arginine at position 4 to nonpolar alanine (R4A) disrupts T-cell recognition for this sequence by all 7 of the 5T4_p17_-specific TCRs (Fig. 3b). The R1A, the arginine to alanine changes at position 1 (R1A) differentially preserved 5T4_p17_-specific TCR recognition. Three of the seven 5T4_p17_ specific TCRs (HD_A-2, HD_A-15, and HD_B-21) killed T2 targets pulsed with R1A at high peptide concentration (Fig. 3b).

## 5T4 _p17_-specific TCR transduced CD8^+^ T-cells lyse 5T4^+^/HLA-A2^+^ tumor targets

**Fig. 4:**
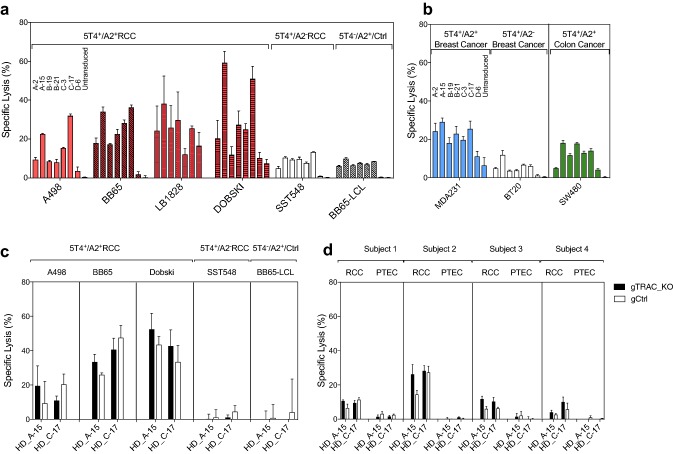
Cytotoxicity of 5T4_p17_-specific TCR-transduced CD8^+^ T-cells against 5T4-expressing tumor targets. CD8^+^ T-cells expressing 5T4_17–25_-specific TCRs were tested for recognition of tumor target lines in a 4-h cytotoxicity assay with 10:1 E:T. For each target cell-line, 5T4_17–25_-specific TCR transduced effector CD8^+^ T-cells were plotted in the following order: HD_A-2, HD_A-15, HD_B-19, HD_B-21, HD_C-3, HD_C-17, KCD_D-6, and untransduced. **a** Targets are 5T4^+^/HLA-A2^+^ RCC cell lines (A498, BB65, TREP, DOBSKI; red bars), the 5T4^+^/HLA-A2^−^ RCC line (SST548; open bars) and the 5T4^−^/HLA-A2^+^ LCL (BB65-LCL; gray shading). **b** Targets are 5T4^+^/HLA-A2^+^ breast cancer line (MDA231; blue bars), a 5T4^+^/HLA-A2^−^ breast cancer line (BT20; open bars) and 5T4^+^/HLA-A2^+^ colon cancer line (SW480, green bars). CD8^+^ T-cells with (gTRAC_KO) or without (gCtrl) native *TRA* disruption expressing 5T4 _p17_-specific TCRs were tested for recognition of **c** 5T4^+^/HLA-A2^+^ RCC cell-lines (A498, BB65, DOBSKI), the 5T4^+^/HLA-A2^−^ RCC line (SST548), the 5T4^−^/HLA-A2^+^ BB65-LCL, and **d** paired primary 5T4^+^/HLA-A2^+^ RCC and autologous PTEC cell-lines from four RCC patients

We tested the cytotoxicity of 5T4_p17_-specific TCR**-**expressing T-cells against 5T4^+^/HLA-A2^+^ RCC (A498, BB65, LB1828, DOBSKI), breast cancer (MDA231) and a colon cancer-cell line (SW480) versus HLA-mismatched (SST548, BT20) or 5T4-negative target-cells (BB65-LCL). T-cells expressing each of the seven 5T4_p17_-specific TCRs demonstrated specific lysis above background levels with control targets for at least one 5T4^+^/HLA-A2^+^ tumor line (Fig. 4a, b). Among the seven TCRs, T-cells expressing HD_A-15 and HD_C-17 consistently had the highest lytic potency against all 5T4^+^/HLA-A2^+^ targets tested including RCC, breast cancer, and colon cancer-cell lines.

In some experiments, low-level lysis by 5T4 TCR-transduced T-cells was observed with HLA-mismatched (SST548, BT20) or 5T4-negative target-cells (BB65-LCL) versus control-target cell lysis seen with the originating T-cell clones. We speculated that low-level cytolytic activity for control-target cells could represent alloreactivity of the heterogeneous endogenous TCR repertoire expressed on the activated, CD8^+^ effector population transduced with 5T4-specific TCRs. To test this hypothesis, we transiently introduced CRISPR/Cas9 guide-RNA targeting of endogenous *TRAC* by electroporation into healthy donor CD8^+^ T-cells. CRISPR/Cas9 mediated *TRAC* targeting disrupted native TCR expression in 97.8% of electroporated T-cells indicated by loss of surface CD3 protein expression (Supplementary Fig. 4a, d). These CD3-negative T-cells were then transduced with lentiviral vectors directing 5T4-specific TCR expression. Residual CRISPR/Cas9 activity was not anticipated to interfere with 5T4-TCR expression due to nucleotide changes in *TRAC* resulting from codon optimization of the 5T4-TCR sequences. The efficiency of lentiviral transduction and surface level of 5T4-specific TCRs remained unchanged compared with control electroporated T-cells (Supplementary Fig. 4b, c, e, f).

Transduced CD8^+^ T-cells with or without native *TRAC* disruption expressing HD_A-15 or HD_C-17 TCRs were re-tested for recognition of 5T4^+^/HLA-A2^+^ RCC (A498, BB65, DOBSKI) versus control targets demonstrating preserved lytic potency for the 5T4^+^/HLA-A2^+^ RCC lines and absent recognition of the control targets, including the HLA-mismatched SST548 RCC or 5T4-negative targets BB65-LCL and HLA-A2^+^/5T4^−^ fibroblast lines (Fig. 4c and Supplementary Fig. 5). The effector activity profile for 5T4-TCR transduced T-cells with native *TRAC* disruption was indistinguishable from the originating T-cell clones [10].

Transduced CD8^+^ T-cells with or without native *TRAC* disruption expressing HD_A-15 or HD_C-17 TCRs were then tested for recognition of primary 5T4^+^/HLA-A2^+^ RCC tumor cells versus autologous PTEC isolated from 4 donors (Supplementary Fig. 6). 5T4-TCR transduced T-cells with native *TRAC* disruption demonstrated specific tumor lysis for all 4 RCC tumors (5–30% specific lysis) with no lytic activity for the patient-matched syngeneic 5T4^−^ PTEC targets (Fig. 4d and Supplementary Fig. 6).

## 5T4_p17_-specific TCR transduced CD8^+^ T-cells detect transporter associated with antigen processing (TAP)-independent processing of 5T4_p17_ in T2 cells

**Fig. 5:**
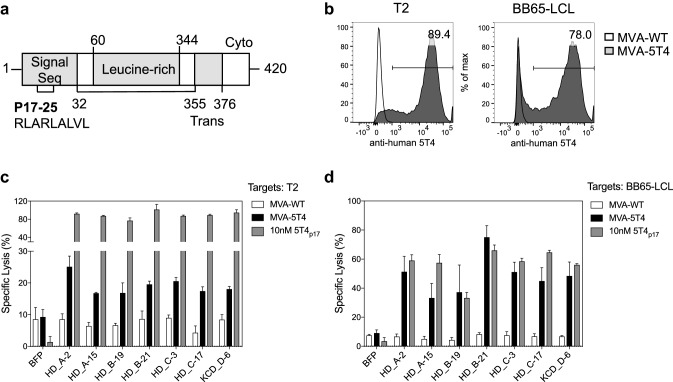
Cytotoxicity of 5T4_p17_-specific TCR-transduced CD8^+^ T-cells against MVA-5T4 infected targets. **a** Schematic of human 5T4 protein, p17–25 is in the signal sequence region. Trans: transmembrane region, Cyto: cytoplasmic region. **b** T2 cells and BB65-LCL were infected with MVA-WT or MVA-5T4, and cell surface expression of 5T4 was analyzed by immunostaining and flow cytometry 24 h after infection. The fraction of cells positive for cell surface 5T4 after MVA-5T4 infection is indicated. CD8^+^ T-cells expressing 5T4_p17_-specific TCRs were tested for recognition of **c** T2 and **d** BB65-LCL cells infected by MVA-WT, MVA-5T4, or uninfected cells pulsed with 10 nM 5T4_p17_ peptide in a 6-h cytotoxicity assay at a 10:1 E:T

One unique characteristic of 5T4_p17_ is its location within the signal sequence of 5T4 (Fig. 5a). Prior studies have shown that some T-cell epitopes within leader domains can load MHC-class I via a TAP-independent pathway as an alternative to the classic antigen presentation pathway mediated by proteasome degradation and TAP transport [39]. The T2 cell-line has bi-allelic deletions of chromosome 6 spanning the *TAP1/2* genes [40]. Therefore, we tested the cytotoxicity of 5T4_p17_-specific TCR expressing T-cells against the T2 cell-line (HLA-A2^+^) versus a wild-type LCL target-line (BB65-LCL, HLA-A2^+^). Both T2 cells and BB65-LCLs express comparable 5T4 protein on their surface when infected with a recombinant vaccinia virus encoding the full-length 5T4 gene (MVA-5T4, Fig. 5b). 5T4_p17_-specific T-cells consistently recognized and lysed MVA-5T4 infected T2 cells above the background killing of MVA-WT infected-T2 targets (Fig. 5c), suggesting the presence of TAP1/2-independent presentation of the 5T4_p17_ epitope in T2 cells. The specific lysis for 5T4-expressing T2 targets was always less than for 5T4-expressing BB65-LCL (Fig. 5d). These results suggest that in the antigen processing-competent target-cells, 5T4_p17_-processing and MHC-class I presentation may result from both the classical processing pathway as well as by a TAP-independent mechanism.

## Discussion

In this report, we have identified 7 unique TCRs with specificity for the 5T4_p17_ epitope presented by HLA-A2. Our analysis of the *TRA* and *TRB* gene segment usage, as well as TRA-CDR3 amino acid sequences, identified a high degree of homology between TCRs. We identified clones with shared *V*-and *J*-gene segments isolated from unrelated donors for both *TRA* and *TRB*. Three similar α-chains from two donors (KCD_D-6, HD_C-3, and HD_C-17) utilized the same *TRAV38*-*2/TRAJ45* and *TRBV6*-*3*, differing by only one amino acid within TRA-CDR3. It has been shown that TCRs against the same tumor-associated epitope use biased sets of variable gene segments. For example, CD8^+^ T-cells in patients and healthy donors specific for an HLA-A2 associated Wilms’ tumor 1 epitope (WT1_126–134_) shared the usage of *TRBV3, 6, 7, 20, 27* [41]; and CD8^+^ T-cell clones specific for the HLA-A2 associated Melanoma antigen recognized by T-cells 1 (MART-1_26–35_) epitope used *TRAV12*-*2* [42]. These similarities reflect the convergent nature of peptide/MHC-TCR binding in different individuals likely as a result of physical constraints on TCR sequence to generate high avidity binding to the peptide/MHC complex. We speculate that interactions between a negatively-charged CDR3 region with the positively charged 5T4_p17_ is a key contributor to the TCR recognition of the 5T4_p17_ epitope sequence.

To evaluate the suitability of 5T4_p17_-specific TCRs for T-cell therapy, we assessed the expression profile, antigen specificity, lytic potency, and off-target reactivity for the seven TCRs. Each TCR efficiently assembles in transduced-healthy CD8^+^ T-cells, is readily detectable at the cell surface, and redirects potent T-cell effector functions including cytotoxicity and cytokine release for HLA-A2^+^-target cells pulsed with 5T4_p17_. Differences in avidity between these TCRs were distinguished when 5T4_p17_-specific TCR-transduced CD8^+^ T-cells were tested against limiting concentration of 5T4_p17_ revealing 10-fold lower avidity for the HD_B-19 TCR versus the other sequences. The specificity of the TCR’s also differed for recognition of an alanine-substituted version of 5T4_p17_ at position 1 (R1A) recognizable by only three of the seven TCRs (HD_A-15, HD_C-17, and HD_B-21). Most importantly, the cytolytic activity of 5T4_p17_-specific TCR transduced CD8^+^ T-cells was demonstrated with a panel of 5T4^+^/HLA-A2^+^ tumor targets including RCC, breast, and colon cancer tumor lines and primary RCC tumor in short-term culture. T-cells transduced with HD_A-15 or HD_C-17 TCRs demonstrated the highest and most consistent specific lysis for the tumor lines tested.

An NCBI peptide blast search reveals that the 9-mer 5T4_p17–25_ sequence is unique to 5T4 in the human peptidome. The 8-mer 5T4_p18–25_ sequence is also unique to 5T4, indicating a potential cross-reactivity of R1A 5T4_p17–25_ specific TCRs for a heterologous protein containing a shared 5T4_p18–25_ sequence is not expected. In performing functional testing of 5T4_p17_-specific TCR transduced CD8^+^ T-cells, we noted low-level cytolytic activity for 5T4^−^ or HLA-A2^−^ target cells that had not been observed with the originating T-cell clones. Using CRISPR/Cas9 mediated *TRAC* targeting and disruption of native TCR expression, we provide direct evidence that allo-reactivity of the heterogenous endogenous TCR repertoire expressed on the activated, CD8^+^ effector population transduced with 5T4-specific TCRs accounts for 5T4 independent-target reactivity. 5T4-TCR transduced T-cells with native *TRAC* disruption showed no lytic activity for 5T4-negative cells representing healthy tissues that included LCL, fibroblast, and PTEC targets. *TRAC* disruption may also prevent the risk of TCR chain mispairing that could result in unexpected auto-reactivity [43]. Whether disruption of native TCR expression in transduced T-cells could augment specific antigen recognition of transduced TCRs by eliminating competition for TCR signaling co-factors is an area of ongoing investigation.

We searched a database of all publicly available *TRB*-*CDR3* sequences curated by our research group (currently > 320 million unique *TRB*-*CDR3s*) for 5T4_p17_-associated *TRB*-*CDR3* sequences. All seven of the *TRB*-*CDR3s* sequences from 5T4_p17_ specific TCRs were identified in non-RCC bearing individuals. In one public dataset of *TRB*-*CDR3* sequencing from 678 healthy individuals [44], identical *TRB*-*CDR3* sequences to HD_A-15 and HD_C-17 were found in 44 (12 HLA-A2^+^) and 5 (1 HLA-A2^+^) healthy bone marrow samples, respectively. The natural occurrence of these *TRB*-*CDR3s* in HLA-A2^+^ healthy donors suggests the possibility for 5T4-reactive TCRs to circulate in the repertoire of a subset of healthy donors in addition to the individuals providing research samples for our original T-cell culture experiments.

Our sequence analyses have demonstrated that naturally occurring 5T4_p17_-specific T-cells are present at very low-frequency. Our studies set an estimate of precursor frequency for these T-cells as low as 1 in 10^7^ CD8^+^ T-cells in responding donors [10]. These data are consistent with immune monitoring studies performed with MVA-5T4 vaccination that have also revealed a heterogenous response phenotype for induced 5T4-specific T-cell immunity and low titer of measured T-cell responses [13]. Inadequate T-cell priming and expansion likely accounts for the failure of the tumor antigen-specific cancer vaccines MVA-5T4 and IMA901 to show improved survival for RCC patients in phase III clinical trials [45, 46].

In contrast to 5T4-specific vaccination, engineered autologous T-cells expressing CARs or TCRs specific for 5T4 represents an emerging therapy platform that, in principle, will facilitate a massive expansion of tumor-reactive T-cells in treated subjects. 5T4 is a transmembrane antigen and can, therefore, be targeted by either CAR or TCR-based strategies. Preclinical assessment of 5T4-CARs has demonstrated recognition of human nasopharyngeal carcinoma in vitro [8] and ovarian cancer in a murine xenograft model [47]. A potential advantage for TCR targeting of the 5T4_p17_ epitope is its location within the signal sequence of the 5T4 protein. It has been well established that a subset of T-cell epitopes within leader domain sequences can load class I MHC by a TAP-independent processing pathway [39]. Deficient TAP expression is a common tumor-associated phenotype thought to contribute to tumor escape from immune surveillance [48, 49]. We observed specific lysis of MVA-5T4 infected, TAP-deficient T2-target cells above background levels with 5T4_p17_-specific TCR-transduced CD8^+^ T-cells indicating the 5T4_p17_ epitope can be processed by a TAP-independent pathway. The robust specific lysis of 5T4_p17_-specific TCR-transduced CD8^+^ T-cells for MVA-5T4 infected LCL targets also suggests the intriguing clinical scenario that subjects treated with TCR-engineered 5T4_p17_-specific T-cells could be subsequently vaccinated with MVA-5T4 to re-stimulate the transduced effector T-cells against the 5T4 target in vivo.

In summary, TCR-engineered 5T4_p17_-specific CD8^+^ T-cells are of interest for future testing as a cellular-immunotherapy product in subjects with 5T4^+^ tumors.

## Electronic supplementary material

Below is the link to the electronic supplementary material.

Supplementary material 1 (PDF 804 kb)

## Abbreviations

5T4: Trophoblast glycoprotein, TPBG
ADCs: Antibody–drug conjugates
CDR3: Complementarity-determining region 3
cPPT: Central polypurine tract
CTLA4: Cytotoxic T-lymphocyte associated protein 4
IMGT: International Immunogenetics Information System
MART-1: Melanoma antigen recognized by T-cells 1
MSCV: Murine stem cell virus
mTOR: Mammalian target of rapamycin
MVA: Modified vaccinia virus Ankara
PTEC: Proximal tubule endothelial cell
RCC: Renal cell carcinoma
TAP: Transporter associated with antigen processing
TET: Tetramer
TKIs: Tyrosine kinase inhibitors
TNF-α: Tumor necrosis factor-α
TRA: T-cell receptor α chain
TRA-CDR3: CDR3 region from TCR-α gene
TRAC: Constant region of TCR-α gene
TRB: T-cell receptor β chain
TRB-CDR3: CDR3 regions from the TCR-β gene
TRBC: Constant region of TCR-β gene
WT1: Wilms’ tumor 1
WPRE: Woodchuck posttranscriptional regulatory element
