# Supplementary material for: Preclinical development of T-cell receptor-engineered T-cell therapy targeting the 5T4 tumor antigen on renal cell carcinoma
Source: Cancer Immunol Immunother. 2019 Nov 4;68(12):1979–93. doi: 10.1007/s00262-019-02419-4 (PMC6877496; doi:10.1007/s00262-019-02419-4)
Supplement: Supplementary file 1 — Supplementary material 1 (PDF 804 kb) [file 262_2019_2419_MOESM1_ESM.pdf]

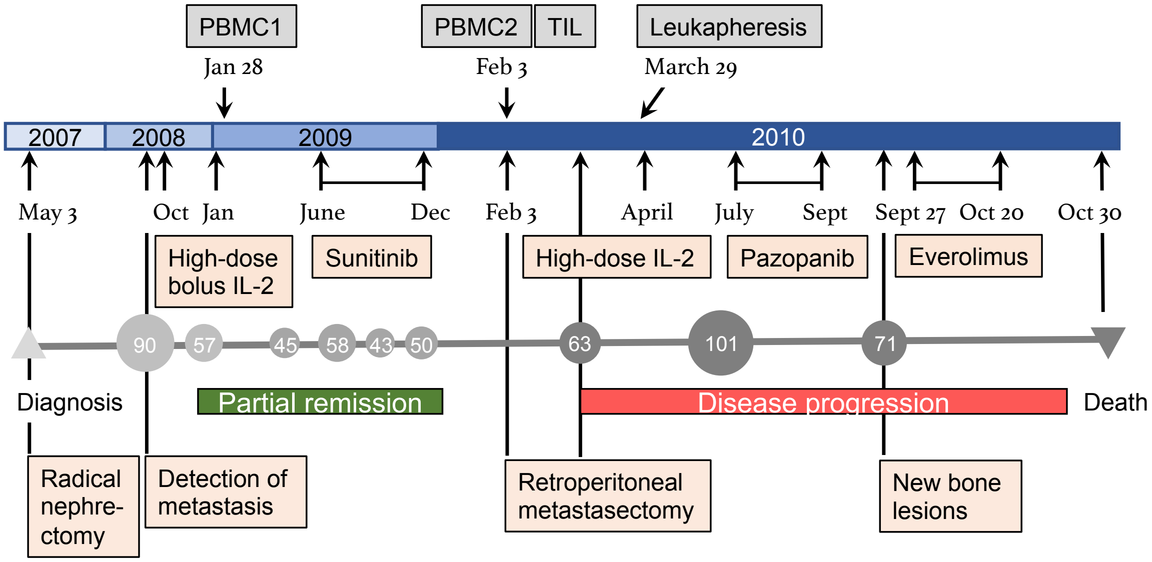

Supplementary Fig. 1 Sample collection and clinical course for kidney cancer donor D (KCD_D). A 56-year old man underwent left nephrectomy surgery and was diagnosed with stage III clear cell renal cell carcinoma. Seventeen months later, he progressed with metastatic disease involving lungs, left nephrectomy bed, and mediastinal and retroperitoneal lymph nodes. Sequential systemic therapies are indicated. After two courses of high dose IL-2 followed by sunitinib, the only remaining metastatic site in the left nephrectomy bed was resected. The patient then progressed shortly thereafter. Disease burden (in mm) assessed from CT body imaging according to RECIST1.1 criteria is shown in gray circles. The collection of peripheral blood mononuclear cell (PBMC), leukapheresis, or tumor-infiltrating lymphocyte (TIL) samples relative to the clinical course are indicated at the top of the figure.

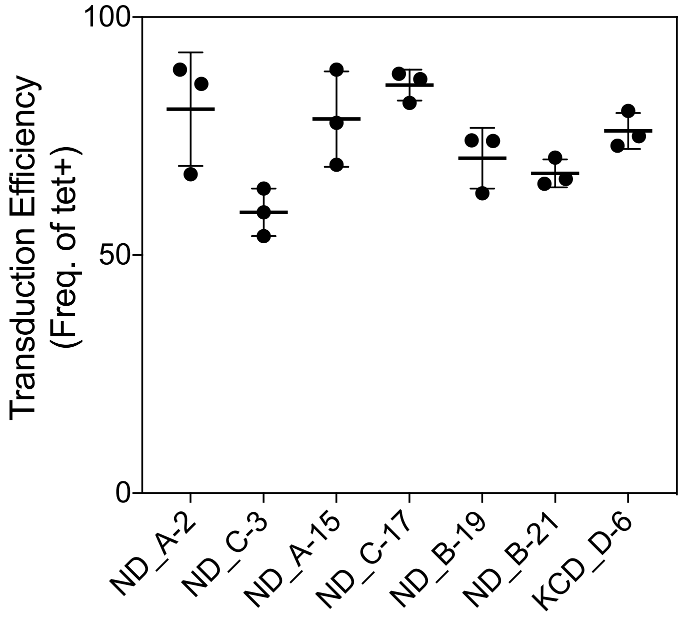

Supplementary Fig. 2 $5T4_{p17}$-specific TCR expression on $CD8^+$ T-cells from healthy donors. The percent of $CD8^+$ T-cells expressing $5T4_{p17}$-specific TCRs stained by $5T4_{p17}$/HLA-A2 tetramer at day 7 post transduction is shown for three different healthy donors.

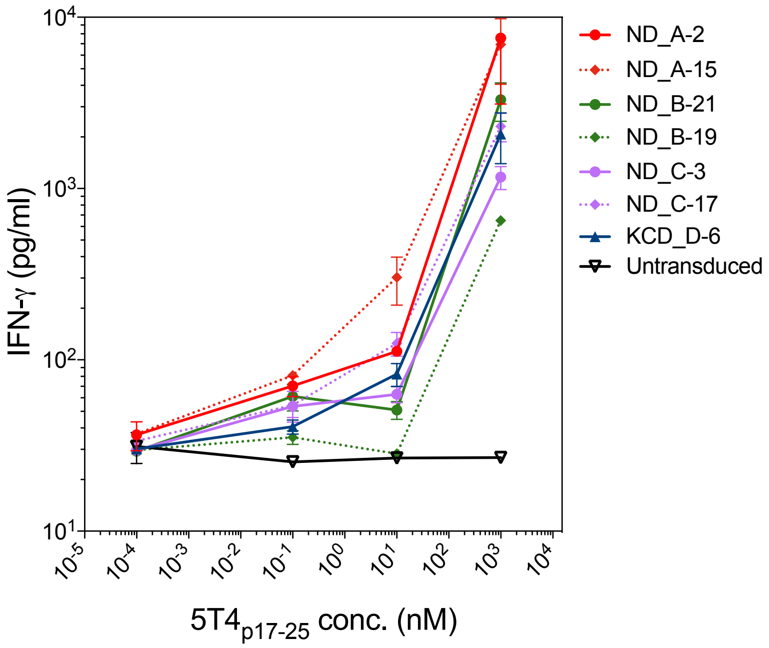

Supplementary Fig. 3 IFN- *γ* release by $5T4_{p17}$-specific TCR transduced $CD8^+$ T-cells stimulated with peptide pulsed T2 targets. IFN-*γ* release was measured by ELISA in culture supernatants harvested after 18 hours co-culture of $CD8^+$ T-cells expressing $5T4_{p17}$-specific TCRs with T2 cells pulsed with $5T4_{p17}$ peptide at 10:1 E : T.

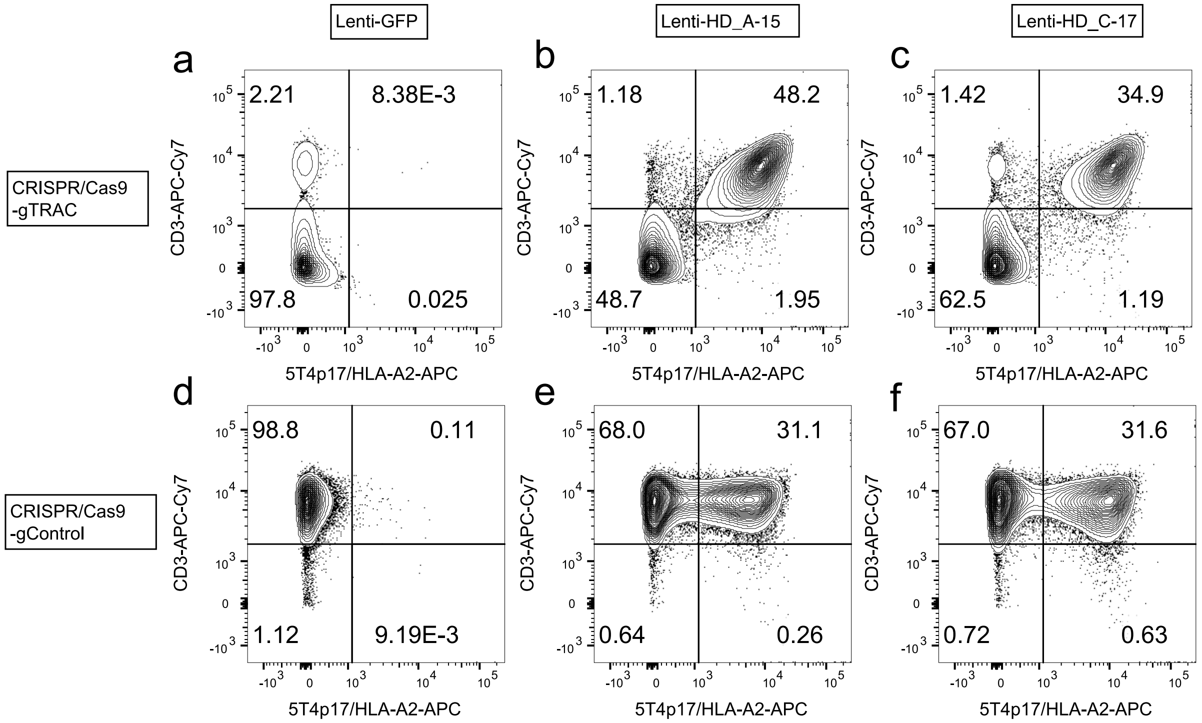

Supplementary Fig. 4 Flow cytometry analysis of endogenous TCR knockout and 5T4 specific TCR expression in healthy donor CD8$^+$ T-cells. T-cells were electroporated with CRISPR/Cas9-gRNA complex targeting the constant region of the *TRA* locus (upper panel), or safe harbor control RNA (lower panel). At day 5 post electroporation, endogenous TCR disruption was confirmed by the loss of surface expression of CD3 compared with control gRNA (a and d). Re-expression of transduced HD_A-15 and HD_C-17 TCRs following endogenous TCR knock out was confirmed with 5T4$_{p17}$/HLA-A2-tetratmer immunostaining (b and c).

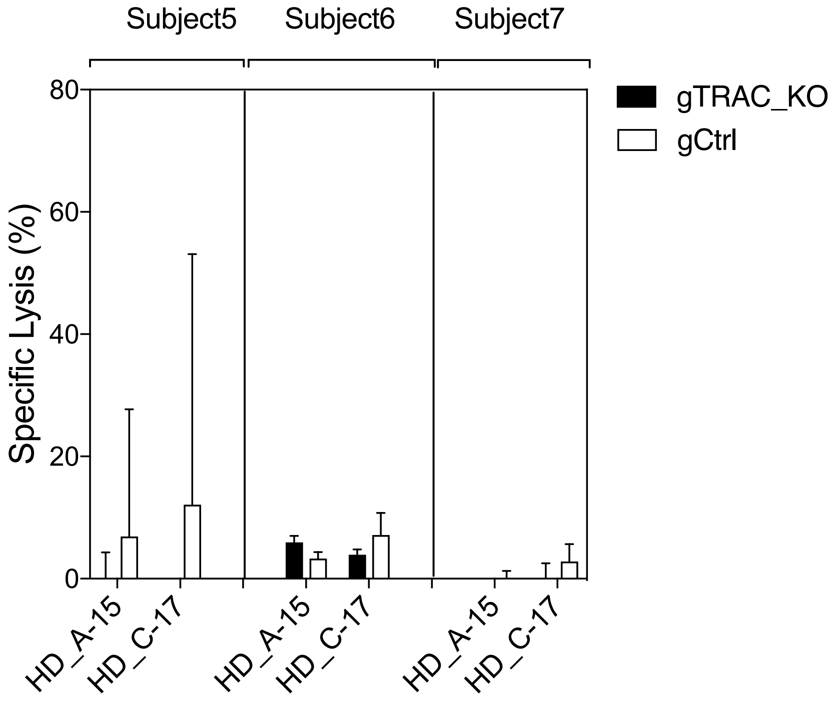

Supplementary Fig. 5 Cytotoxicity of $5T4_{p17}$-specific TCR transduced $CD8^+$ T-cells with or without endogenous TRA disruption against $HLA\text{-}A2^+$ fibroblast targets.

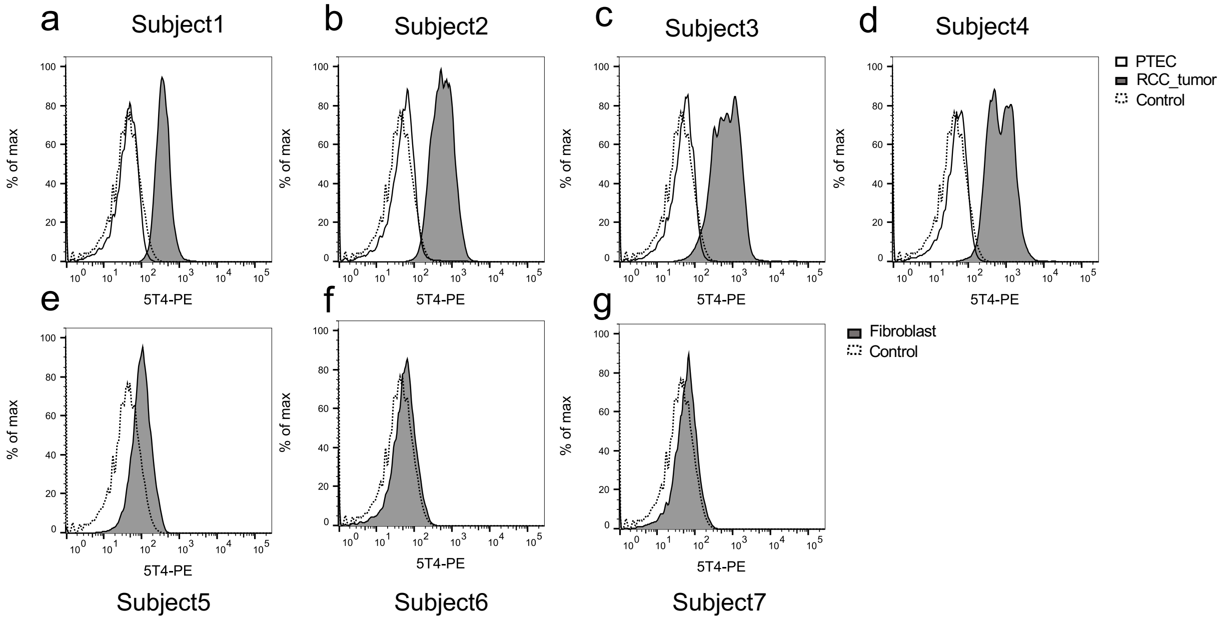

Supplementary Fig. 6 Flow cytometry analysis of cell surface 5T4 expression. Primary RCC cell lines from four HLA-A2$^+$ patients with clear cell RCC tumors and corresponding autologous PTEC lines were immunostained for 5T4 expression and analyzed by flow cytometry (a-d). Dermal fibroblast lines from three RCC patients (unrelated to donors for samples a-d) were also immunostained for 5T4 expression (e-g). RCC-tumor line stained with secondary PE-conjugated antibody was used as control.

Supplementary Table 1. 5T4$_{p17}$-specific *TRB-CDR3* sequencing

Donor	Tissue	Unique *TRB-CDR3*	Estimated Genomes
HD_A	Leukapheresis	11232	147074
HD_B	Leukapheresis	7155	8303
KCD_D	Leukapheresis	25407	147599
KCD_D	PBMC	5888	21834
KCD_D	PBMC	19020	79359
KCD_D	TIL	1332	2158

Abbreviations: TRB, T-cell receptor beta; CDR3, complimentarity determining region 3

Supplementary Table 2. Amino acid sequences for CDR1-3 domains from 5T4$_{p17}$-specific TCRs

Clone	CDR1α	CDR2α	CDR3α	CDR1β	CDR2β	CDR3β
HD_A-2	DRGSQS	IYSNGD	CAVPDDAGNMLTF	WSHSY	SAAADI	CASSELPAGGTNEQFF
HD_A-15	TSENNYY	QEAYKQQ	CASMYSGGGADGLTF	MNHEY	SMNVEV	CASSFFSNTGELFF
KCD_D-6	TSESDYY	QEAYKQQ	CASGGGADGLTF	MNHEY	SVGEGT	CASSFLTDTQYF
HD_C-17	TSESDYY	QEAYKQQ	CAGGGGADGLTF	MNHEY	SVGEGT	CASSYMGPEAFF
HD_C-3	TSESDYY	QEAYKQQ	CSSGGGADGLTF	MNHEY	SVGEGT	CASMDLAFKQYF
HD_B-19	TSESDYY	QEAYKQQ	CAYRSGSDGGSQGNLIF	LGHNA	YSLEER	CASSQVSGYEQYF
HD_B-21	TSGFYG	NALDG	CAVRDDYGQNFVF	KGHSH	LQKEN	CASSPQGDNEQFF

Supplementary Table 3. Amino acid sequences of 5T4-specific TCRs

Clone	TRA	TRB
ND_A-2	MKSLRVLLVILWLQLSWVWSQQKEVEQNSGPLSVPEGAIASLNCTYSD<br>RGSQSFFWYRQYSGKSPELIMFIYSNGDKEDGRFTAQLNKASQYVSLL<br>IRDSQPSDSATYLCAVPDDAGNMLTFGGGTRLMVKPHDIQNPDPAVYQ<br>LRDSKSSDKSVCLFTDFDSQTNVSQSKDSDVYITDKCVLDMRSMDFKS<br>NSAVAWSNKSDFACANAFNNSIIPEDTFFPSPESSCDVKLVEKSFETD<br>TNLNFQNLSVIGFRILLLKVAGFNLLMTLRLWSS	MGTRLFFYVALCLLWAGHRDAGITQSPRYKITETGRQVTLMCHQT<br>WSHSYMFWYRQDLGHGLRLIYYSAAADITDKGEVPDGYVVSRSKT<br>ENFPLTLESATRSQTSVYFCASSELPAGGTNEQFFGPGTRLTVLD<br>LKNVFPPEVAVFEPSEAEISHTQKATLVCLATGFFPDHVELSWWV<br>NGKEVHSGVCTDPQPLKEQPALNDSRYCLSSRLRVSATFWQNPRN<br>HFRCQVQFYGLSENDEWTQDRAKPVTQIVSAEAWGRADCGFTSVS<br>YQQGV
ND_C-3	MACPGFLWALVISTCLEFSMAQTVTQSQPEMSVQEAETVTLSCTYDTS<br>ESDYYLFWYKQPPSRQMILVIRQEAYKQQNATENRFSVNFQKAAKSFS<br>LKISDSQLGDAAMYFCSSGGGADGLTFGKGTHLIIQPYDIQNPDPAVY<br>QLRDSKSSDKSVCLFTDFDSQTNVSQSKDSDVYITDKCVLDMRSMDFK<br>SNSAVAWSNKSDFACANAFNNSIIPEDTFFPSPESSCDVKLVEKSFET<br>DTNLNFQNLSVIGFRILLLKVAGFNLLMTLRLWSS	MSLGLLCCGAFSLLWAGPVNAGVTQTPKFRVLKTGQSMTLLCAQD<br>MNHEYMYWYRQDPGMGLRLIHYSVGEGTTAKGEVPDGYNVSRLKK<br>QNFLLGLESAAPSQTSVYFCASMDLAFKQYFGPGTRLTVTEDLKN<br>VFPPEVAVFEPSEAEISHTQKATLVCLATGFFPDHVELSWWVNGK<br>EVHSGVCTDPQPLKEQPALNDSRYCLSSRLRVSATFWQNPRNHFR<br>CQVQFYGLSENDEWTQDRAKPVTQIVSAEAWGRADCGFTSVSYQQ<br>GVLSATILYEILLGKATLYAVLVSALVLMAMVKRKDF
KCD_D-6	MACPGFLWALVISTCLEFSMAQTVTQSQPEMSVQEAETVTLSCTYDTS<br>ESDYYLFWYKQPPSRQMILVIRQEAYKQQNATENRFSVNFQKAAKSFS<br>LKISDSQLGDAAMYFCASGGGADGLTFGKGTHLIIQPYDIQNPDPAVY<br>QLRDSKSSDKSVCLFTDFDSQTNVSQSKDSDVYITDKCVLDMRSMDFK<br>SNSAVAWSNKSDFACANAFNNSIIPEDTFFPSPESSCDVKLVEKSFET<br>DTNLNFQNLSVIGFRILLLKVAGFNLLMTLRLWSS	MSLGLLCCGAFSLLWAGPVNAGVTQTPKFRVLKTGQSMTLLCAQD<br>MNHEYMYWYRQDPGMGLRLIHYSVGEGTTAKGEVPDGYNVSRLKK<br>QNFLLGLESAAPSQTSVYFCASSFLTDTQYFGPGTRLTVLEDLKN<br>VFPPEVAVFEPSEAEISHTQKATLVCLATGFFPDHVELSWWVNGK<br>EVHSGVCTDPQPLKEQPALNDSRYCLSSRLRVSATFWQNPRNHFR<br>CQVQFYGLSENDEWTQDRAKPVTQIVSAEAWGRADCGFTSVSYQQ<br>GVLSATILYEILLGKATLYAVLVSALVLMAMVKRKDF
ND_A-15	MTRVSLLWAVVVSTCLESGMAQTVTQSQPEMSVQEAETVTLSCTYDTS<br>ENNYYLFWYKQPPSRQMILVIRQEAYKQQNATENRFSVNFQKAAKSFS<br>LKISDSQLGDTAMYFCASMYSGGGADGLTFGKGTHLIIQPYDIQNPDP<br>AVYQLRDSKSSDKSVCLFTDFDSQTNVSQSKDSDVYITDKCVLDMRSM<br>DFKSNSAVAWSNKSDFACANAFNNSIIPEDTFFPSPESSCDVKLVEKS<br>FETDTNLNFQNLSVIGFRILLLKVAGFNLLMTLRLWSS	MGPQLLGYVVLCLLGAGPLEAQVTQNPRYLITVTGKKLTVTCSQN<br>MNHEYMSWYRQDPGLGLRQIYYSMNVEVTDKGDVPEGYKVSRKEK<br>RNFPLILESPSPNQTSLYFCASSFFSNTGELFFGEGSRLTVLEDL<br>NKVFPPEVAVFEPSEAEISHTQKATLVCLATGFFPDHVELSWWVN<br>GKEVHSGVCTDPQPLKEQPALNDSRYCLSSRLRVSATFWQNPRNH<br>FRCQVQFYGLSENDEWTQDRAKPVTQIVSAEAWGRADCGFTSVSY<br>QQGVLSATILYEILLGKATLYAVLVSALVLMAMVKRKDF
ND_C-17	MACPGFLWALVISTCLEFSMAQTVTQSQPEMSVQEAETVTLSCTYDTS<br>ESDYYLFWYKQPPSRQMILVIRQEAYKQQNATENRFSVNFQKAAKSFS<br>LKISDSQLGDAAMYFCAGGGGADGLTFGKGTHLIIQPYDIQNPDPAVY<br>QLRDSKSSDKSVCLFTDFDSQTNVSQSKDSDVYITDKCVLDMRSMDFK<br>SNSAVAWSNKSDFACANAFNNSIIPEDTFFPSPESSCDVKLVEKSFET<br>DTNLNFQNLSVIGFRILLLKVAGFNLLMTLRLWSS	MSLGLLCCGAFSLLWAGPVNAGVTQTPKFRVLKTGQSMTLLCAQD<br>MNHEYMYWYRQDPGMGLRLIHYSVGEGTTAKGEVPDGYNVSRLKK<br>QNFLLGLESAAPSQTSVYFCASSYMGPEAFFGQGTRLTVVEDLNK<br>VFPPEVAVFEPSEAEISHTQKATLVCLATGFFPDHVELSWWVNGK<br>EVHSGVCTDPQPLKEQPALNDSRYCLSSRLRVSATFWQNPRNHFR<br>CQVQFYGLSENDEWTQDRAKPVTQIVSAEAWGRADCGFTSVSYQQ<br>GVLSATILYEILLGKATLYAVLVSALVLMAMVKRKDF
ND_B-19	MACPGFLWALVISTCLEFSMAQTVTQSQPEMSVQEAETVTLSCTYDTS<br>ESDYYLFWYKQPPSRQMILVIRQEAYKQQNATENRFSVNFQKAAKSFS<br>LKISDSQLGDAAMYFCAYRSGSDGGSQGNLIFGKGTKLSVKPNDIQNP<br>DPAVYQLRDSKSSDKSVCLFTDFDSQTNVSQSKDSDVYITDKCVLDMR<br>SMDFKSNSAVAWSNKSDFACANAFNNSIIPEDTFFPSPESSCDVKLVE<br>KSFETDTNLNFQNLSVIGFRILLLKVAGFNLLMTLRLWSS	MGCRLLCCAVLCLLGAGELVPMETGVTQTPRHLVMGMTNKKSLKC<br>EQHLGHNAMYWYKQSAKKPLELMFVYSLEERVENNSVPSRFSPEC<br>PNSSHLFLHLHTLQPEDSALYLCASSQVSGYEQYFGPGTRLTVTE<br>DLKNVFPPEVAVFEPSEAEISHTQKATLVCLATGFFPDHVELSWW<br>VNGKEVHSGVCTDPQPLKEQPALNDSRYCLSSRLRVSATFWQNPR<br>NHFRCQVQFYGLSENDEWTQDRAKPVTQIVSAEAWGRADCGFTSV<br>SYQQGVLSATILYEILLGKATLYAVLVSALVLMAMVKRKDF
ND_B-21	MWGAFLLYVSMKMGGTAGQSLEQPSEVTAVEGAIVQINCTYQTSGFYG<br>LSWYQQHDGGAPTFLSYNALDGLEETGRFSSFLSRSDSYGYLLLQELQ<br>MKDSASYFCAVRDDYGQNFVFGPGTRLSVLPYDIQNPDPAVYQLRDSK<br>SSDKSVCLFTDFDSQTNVSQSKDSDVYITDKCVLDMRSMDFKSNSAVA<br>WSNKSDFACANAFNNSIIPEDTFFPSPESSCDVKLVEKSFETDTNLNF<br>QNLSVIGFRILLLKVAGFNLLMTLRLWSS	MDTRVLCCAVICLLGAGLSNAGVMQNPRHLVRRRGQEARLRCSPM<br>KGHSHVYWYRQLPEEGLKFMVYLQKENIIDESGMPKERFSAEFPK<br>EGPSILRIQQVVRGDSAAYFCASSPQGDNEQFFGPGTRLTVLEDL<br>NKVFPPEVAVFEPSEAEISHTQKATLVCLATGFFPDHVELSWWVN<br>GKEVHSGVCTDPQPLKEQPALNDSRYCLSSRLRVSATFWQNPRNH<br>FRCQVQFYGLSENDEWTQDRAKPVTQIVSAEAWGRADCGFTSVSY<br>QQGVLSATILYEILLGKATLYAVLVSALVLMAMVKRKDF

Supplementary Table 4. Native nucleotide sequences of 5T4-specific TCRs

Clone	TRA	TRB
ND_A-2	ATGAAATCCTTGAGAGTTTTACTAGTGATCCTGTGGCTTCAGTTGAGCTGGGTTTGGAGCCAACAGAAGGAGGTG<br>GAGCAGAATTCTGGACCCCTCAGTGTTCCAGAGGGAGCCATTGCCTCTCTCAACTGCACTTACAGTGACCGAGGT<br>TCCCAGTCCTTCTTCTGGTACAGACAATATTCTGGGAAAAGCCCTGAGTTGATAATGTTCATATACTCCAATGGT<br>GACAAAGAAGATGGAAGGTTTACAGCACAGCTCAATAAAGCCAGCCAGTATGTTTCTCTGCTCATCAGAGACTCC<br>CAGCCCAGTGATTCAGCCACCTACCTCTGTGCCGTCCCGGACGATGCAGGCAACATGCTCACCTTTGGAGGGGGA<br>ACAAGGTTAATGGTCAAACCCCATATCCAGAACCCTGACCCTGCCGTGTACCAGCTGAGAGACTCTAAATCCAGT<br>GACAAGTCTGTCTGCCTATTCACCGATTTTGATTCTCAAACAAATGTGTCACAAAGTAAGGATTCTGATGTGTAT<br>ATCACAGACAAATGTGTGCTAGACATGAGGTCTATGGACTTCAAGAGCAACAGTGCTGTGGCCTGGAGCAACAAA<br>TCTGACTTTGCATGTGCAAACGCCTTCAACAACAGCATTATTCCAGAAGACACCTTCTTCCCCAGCCCAGAAAGT<br>TCCTGTGATGTCAAGCTGGTCGAGAAAAGCTTTGAAACAGATACGAACCTAAACTTTCAAAACCTGTCAGTGATT<br>GGGTTCCGAATCCTCCTCCTGAAAGTGGCCGGGTTTAATCTGCTCATGACGCTGCGGCTGTGGTCCAGCTGA	ATGGGCACCAGGCTCTTCTTCTATGTGGCCCTTTGTCTGCTGTGGGCAGGACACAGGGATGCTGGAATCACCCAGAGCCCAAGAT<br>ACAAGATCACAGAGACAGGAAGGCAGGTGACCTTGATGTGTCACCAGACTTGGAGCCACAGCTATATGTTCTGGTATCGACAAGA<br>CCTGGGACATGGGCTGAGGCTGATCTATTACTCAGCAGCTGCTGATATTACAGATAAAGGAGAAGTCCCCGATGGCTATGTTGTC<br>TCCAGATCCAAGACAGAGAATTTCCCCCTCACTCTGGAGTCAGCTACCCGCTCCCAGACATCTGTGTATTTCTGCGCCAGCAGTG<br>AGCTCCCAGCGGGAGGGACGAATGAGCAGTTCTTCGGGCCAGGGACACGGCTCACCGTGCTAGACCTGAAAAACGTGTTCCCACC<br>CGAGGTCGCTGTGTTTGAGCCATCAGAAGCAGAGATCTCCCACACCCAAAAGGCCACACTGGTGTGCCTGGCCACAGGCTTCTAC<br>CCCGACCACGTGGAGCTGAGCTGGTGGGTGAATGGGAAGGAGGTGCACAGTGGGGTCTGCACAGACCCGCAGCCCCTCAAGGAGC<br>AGCCCGCCCTCAATGACTCCAGATACTGCCTGAGCAGCCGCCTGAGGGTCTCGGCCACCTTCTGGCAGAACCCCCGCAACCACTT<br>CCGCTGTCAAGTCCAGTTCTACGGGCTCTCGGAGAATGACGAGTGGACCCAGGATAGGGCCAAACCTGTCACCCAGATCGTCAGC<br>GCCGAGGCCTGGGGTAGAGCAGACTGTGGCTTCACCTCCGAGTCTTACCAGCAAGGGGTCCTGTCTGCCACCATCCTCTATGAGA<br>TCTTGCTAGGGAAGGCCACCTTGTATGCCGTGCTGGTCAGTGCCCTCGTGCTGATGGCCATGGTCAAGAGAAAGGATTCCAGAGG<br>CTAG
ND_C-3	ATGGCATGCCCTGGCTTCCTGTGGGCACTTGTGATCTCCACCTGTCTTGAATTTAGCATGGCTCAGACAGTCACT<br>CAGTCTCAACCAGAGATGTCTGTGCAGGAGGCAGAGACCGTGACCCTGAGCTGCACATATGACACCAGTGAGAGT<br>GATTATTATTTATTCTGGTACAAGCAGCCTCCCAGCAGGCAGATGATTCTCGTTATTCGCCAAGAAGCTTATAAG<br>CAACAGAATGCAACAGAGAATCGTTTCTCTGTGAACTTCCAGAAAGCAGCCAAATCCTTCAGTCTCAAGATCTCA<br>GACTCACAGCTGGGGGATGCCGCGATGTATTTCTGTTCATCAGGAGGAGGTGCTGACGGACTCACCTTTGGCAAA<br>GGGACTCATCTAATCATCCAGCCCTATATCCAGAACCCTGACCCTGCCGTGTACCAGCTGAGAGACTCTAAATCC<br>AGTGACAAGTCTGTCTGCCTATTCACCGATTTTGATTCTCAAACAAATGTGTCACAAAGTAAGGATTCTGATGTG<br>TATATCACAGACAAATGTGTGCTAGACATGAGGTCTATGGACTTCAAGAGCAACAGTGCTGTGGCCTGGAGCAAC<br>AAATCTGACTTTGCATGTGCAAACGCCTTCAACAACAGCATTATTCCAGAAGACACCTTCTTCCCCAGCCCAGAA<br>AGTTCCTGTGATGTCAAGCTGGTCGAGAAAAGCTTTGAAACAGATACGAACCTAAACTTTCAAAACCTGTCAGTG<br>ATTGGGTTCCGAATCCTCCTCCTGAAAGTGGCCGGGTTTAATCTGCTCATGACGCTGCGGCTGTGGTCCAGCTGA	ATGAGCCTCGGGCTCCTGTGCTGTGGGGCCTTTTCTCTCCTGTGGGCAGGTCCAGTGAATGCTGGTGTCACTCAGACCCCAAAAT<br>TCCGGGTCCTGAAGACAGGACAGAGCATGACACTGCTGTGTGCCCAGGATATGAACCATGAATACATGTACTGGTATCGACAAGA<br>CCCAGGCATGGGGCTGAGGCTGATTCATTACTCAGTTGGTGAGGGTACAACTGCCAAAGGAGAGGTCCCTGATGGCTACAATGTC<br>TCCAGATTAAAAAAACAGAATTTCCTGCTGGGGTTGGAGTCGGCTGCTCCCTCCCAAACATCTGTGTACTTCTGTGCCAGCATGG<br>ATCTAGCGTTTAAGCAGTACTTCGGGCCGGGCACCAGGCTCACGGTCACAGAGGACCTGAAAAACGTGTTCCCACCCGAGGTCGC<br>TGTGTTTGAGCCATCAGAAGCAGAGATCTCCCACACCCAAAAGGCCACACTGGTGTGCCTGGCCACAGGCTTCTACCCCGACCAC<br>GTGGAGCTGAGCTGGTGGGTGAATGGGAAGGAGGTGCACAGTGGGGTCTGCACAGACCCGCAGCCCCTCAAGGAGCAGCCCGCCC<br>TCAATGACTCCAGATACTGCCTGAGCAGCCGCCTGAGGGTCTCGGCCACCTTCTGGCAGAACCCCCGCAACCACTTCCGCTGTCA<br>AGTCCAGTTCTACGGGCTCTCGGAGAATGACGAGTGGACCCAGGATAGGGCCAAACCTGTCACCCAGATCGTCAGCGCCGAGGCC<br>TGGGGTAGAGCAGACTGTGGCTTCACCTCCGAGTCTTACCAGCAAGGGGTCCTGTCTGCCACCATCCTCTATGAGATCTTGCTAG<br>GGAAGGCCACCTTGTATGCCGTGCTGGTCAGTGCCCTCGTGCTGATGGCCATGGTCAAGAGAAAGGATTCCAGAGGCTAG
KCD_D-6	ATGGCATGCCCTGGCTTCCTGTGGGCACTTGTGATCTCCACCTGTCTTGAATTTAGCATGGCTCAGACAGTCACT<br>CAGTCTCAACCAGAGATGTCTGTGCAGGAGGCAGAGACCGTGACCCTGAGCTGCACATATGACACCAGTGAGAGT<br>GATTATTATTTATTCTGGTACAAGCAGCCTCCCAGCAGGCAGATGATTCTCGTTATTCGCCAAGAAGCTTATAAG<br>CAACAGAATGCAACAGAGAATCGTTTCTCTGTGAACTTCCAGAAAGCAGCCAAATCCTTCAGTCTCAAGATCTCA<br>GACTCACAGCTGGGGGATGCCGCGATGTATTTCTGTGCCTCTGGAGGAGGTGCTGACGGACTCACCTTTGGCAAA<br>GGGACTCATCTAATCATCCAGCCCTATATCCAGAACCCTGACCCTGCCGTGTACCAGCTGAGAGACTCTAAATCC<br>AGTGACAAGTCTGTCTGCCTATTCACCGATTTTGATTCTCAAACAAATGTGTCACAAAGTAAGGATTCTGATGTG<br>TATATCACAGACAAATGTGTGCTAGACATGAGGTCTATGGACTTCAAGAGCAACAGTGCTGTGGCCTGGAGCAAC<br>AAATCTGACTTTGCATGTGCAAACGCCTTCAACAACAGCATTATTCCAGAAGACACCTTCTTCCCCAGCCCAGAA<br>AGTTCCTGTGATGTCAAGCTGGTCGAGAAAAGCTTTGAAACAGATACGAACCTAAACTTTCAAAACCTGTCAGTG<br>ATTGGGTTCCGAATCCTCCTCCTGAAAGTGGCCGGGTTTAATCTGCTCATGACGCTGCGGCTGTGGTCCAGCTGA	ATGAGCCTCGGGCTCCTGTGCTGTGGGGCCTTTTCTCTCCTGTGGGCAGGTCCAGTGAATGCTGGTGTCACTCAGACCCCAAAAT<br>TCCGGGTCCTGAAGACAGGACAGAGCATGACACTGCTGTGTGCCCAGGATATGAACCATGAATACATGTACTGGTATCGACAAGA<br>CCCAGGCATGGGGCTGAGGCTGATTCATTACTCAGTTGGTGAGGGTACAACTGCCAAAGGAGAGGTCCCTGATGGCTACAATGTC<br>TCCAGATTAAAAAAACAGAATTTCCTGCTGGGGTTGGAGTCGGCTGCTCCCTCCCAAACATCTGTGTACTTCTGTGCCAGCAGTT<br>TCCTTACAGATACGCAGTATTTTGGCCCAGGCACCCGGCTGACAGTGCTCGAGGACCTGAACAAGGTGTTCCCACCCGAGGTCGC<br>TGTGTTTGAGCCATCAGAAGCAGAGATCTCCCACACCCAAAAGGCCACACTGGTGTGCCTGGCCACAGGCTTCTTCCCCGACCAC<br>GTGGAGCTGAGCTGGTGGGTGAATGGGAAGGAGGTGCACAGTGGGGTCTGCACGGACCCGCAGCCCCTCAAGGAGCAGCCCGCCC<br>TCAATGACTCCAGATACTGCCTGAGCAGCCGCCTGAGGGTCTCGGCCACCTTCTGGCAGAACCCCCGCAACCACTTCCGCTGTCA<br>AGTCCAGTTCTACGGGCTCTCGGAGAATGACGAGTGGACCCAGGATAGGGCCAAACCCGTCACCCAGATCGTCAGCGCCGAGGCC<br>TGGGGTAGAGCAGACTGTGGCTTTACCTCGGTGTCCTACCAGCAAGGGGTCCTGTCTGCCACCATCCTCTATGAGATCCTGCTAG<br>GGAAGGCCACCCTGTATGCTGTGCTGGTCAGCGCCCTTGTGTTGATGGCCATGGTCAAGAGAAAGGATTTCTGA
ND_A-15	ATGACACGAGTTAGCTTGCTGTGGGCAGTCGTGGTCTCCACCTGTCTTGAATCCGGCATGGCCCAGACAGTCACT<br>CAGTCTCAACCAGAGATGTCTGTGCAGGAGGCAGAGACTGTGACCCTGAGTTGCACATATGACACCAGTGAGAAT<br>AATTATTATTTGTTCTGGTACAAGCAGCCTCCCAGCAGGCAGATGATTCTCGTTATTCGCCAAGAAGCTTATAAG<br>CAACAGAATGCAACGGAGAATCGTTTCTCTGTGAACTTCCAGAAAGCAGCCAAATCCTTCAGTCTCAAGATCTCA<br>GACTCACAGCTGGGGGACACTGCGATGTATTTCTGTGCCTCGATGTATTCAGGAGGAGGTGCTGACGGACTCACC<br>TTTGGCAAAGGGACTCATCTAATCATCCAGCCCTATATCCAGAACCCTGACCCTGCCGTGTACCAGCTGAGAGAC<br>TCTAAATCCAGTGACAAGTCTGTCTGCCTATTCACCGATTTTGATTCTCAAACAAATGTGTCACAAAGTAAGGAT<br>TCTGATGTGTATATCACAGACAAATGTGTGCTAGACATGAGGTCTATGGACTTCAAGAGCAACAGTGCTGTGGCC<br>TGGAGCAACAAATCTGACTTTGCATGTGCAAACGCCTTCAACAACAGCATTATTCCAGAAGACACCTTCTTCCCC<br>AGCCCAGAAAGTTCCTGTGATGTCAAGCTGGTCGAGAAAAGCTTTGAAACAGATACGAACCTAAACTTTCAAAAC<br>CTGTCAGTGATTGGGTTCCGAATCCTCCTCCTGAAAGTGGCCGGGTTTAATCTGCTCATGACGCTGCGGCTGTGG<br>TCCAGCTGA	ATGGGCCCCCAGCTCCTTGGCTATGTGGTCCTTTGCCTTCTAGGAGCAGGCCCCCTGGAAGCCCAAGTGACCCAGAACCCAAGAT<br>ACCTCATCACAGTGACTGGAAAGAAGTTAACAGTGACTTGTTCTCAGAATATGAACCATGAGTATATGTCCTGGTATCGACAAGA<br>CCCAGGGCTGGGCTTAAGGCAGATCTACTATTCAATGAATGTTGAGGTGACTGATAAGGGAGATGTTCCTGAAGGGTACAAAGTC<br>TCTCGAAAAGAGAAGAGGAATTTCCCCCTGATCCTGGAGTCGCCCAGCCCCAACCAGACCTCTCTGTACTTCTGTGCCAGCAGTT<br>TCTTCTCGAACACCGGGGAGCTGTTTTTTGGAGAAGGCTCTAGGCTGACCGTACTGGAGGACCTGAACAAGGTGTTCCCACCCGA<br>GGTCGCTGTGTTTGAGCCATCAGAAGCAGAGATCTCCCACACCCAAAAGGCCACACTGGTGTGCCTGGCCACAGGCTTCTTCCCC<br>GACCACGTGGAGCTGAGCTGGTGGGTGAATGGGAAGGAGGTGCACAGTGGGGTCTGCACGGACCCGCAGCCCCTCAAGGAGCAGC<br>CCGCCCTCAATGACTCCAGATACTGCCTGAGCAGCCGCCTGAGGGTCTCGGCCACCTTCTGGCAGAACCCCCGCAACCACTTCCG<br>CTGTCAAGTCCAGTTCTACGGGCTCTCGGAGAATGACGAGTGGACCCAGGATAGGGCCAAACCCGTCACCCAGATCGTCAGCGCC<br>GAGGCCTGGGGTAGAGCAGACTGTGGCTTTACCTCGGTGTCCTACCAGCAAGGGGTCCTGTCTGCCACCATCCTCTATGAGATCC<br>TGCTAGGGAAGGCCACCCTGTATGCTGTGCTGGTCAGCGCCCTTGTGTTGATGGCCATGGTCAAGAGAAAGGATTTCTGA
ND_C-17	ATGGCATGCCCTGGCTTCCTGTGGGCACTTGTGATCTCCACCTGTCTTGAATTTAGCATGGCTCAGACAGTCACT<br>CAGTCTCAACCAGAGATGTCTGTGCAGGAGGCAGAGACCGTGACCCTGAGCTGCACATATGACACCAGTGAGAGT<br>GATTATTATTTATTCTGGTACAAGCAGCCTCCCAGCAGGCAGATGATTCTCGTTATTCGCCAAGAAGCTTATAAG<br>CAACAGAATGCAACAGAGAATCGTTTCTCTGTGAACTTCCAGAAAGCAGCCAAATCCTTCAGTCTCAAGATCTCA<br>GACTCACAGCTGGGGGATGCCGCGATGTATTTCTGTGCTGGGGGGGGAGGTGCTGACGGACTCACCTTTGGCAAA<br>GGGACTCATCTAATCATCCAGCCCTATATCCAGAACCCTGACCCTGCCGTGTACCAGCTGAGAGACTCTAAATCC<br>AGTGACAAGTCTGTCTGCCTATTCACCGATTTTGATTCTCAAACAAATGTGTCACAAAGTAAGGATTCTGATGTG<br>TATATCACAGACAAATGTGTGCTAGACATGAGGTCTATGGACTTCAAGAGCAACAGTGCTGTGGCCTGGAGCAAC<br>AAATCTGACTTTGCATGTGCAAACGCCTTCAACAACAGCATTATTCCAGAAGACACCTTCTTCCCCAGCCCAGAA<br>AGTTCCTGTGATGTCAAGCTGGTCGAGAAAAGCTTTGAAACAGATACGAACCTAAACTTTCAAAACCTGTCAGTG<br>ATTGGGTTCCGAATCCTCCTCCTGAAAGTGGCCGGGTTTAATCTGCTCATGACGCTGCGGCTGTGGTCCAGCTGA	ATGAGCCTCGGGCTCCTGTGCTGTGGGGCCTTTTCTCTCCTGTGGGCAGGTCCAGTGAATGCTGGTGTCACTCAGACCCCAAAAT<br>TCCGGGTCCTGAAGACAGGACAGAGCATGACACTGCTGTGTGCCCAGGATATGAACCATGAATACATGTACTGGTATCGACAAGA<br>CCCAGGCATGGGGCTGAGGCTGATTCATTACTCAGTTGGTGAGGGTACAACTGCCAAAGGAGAGGTCCCTGATGGCTACAATGTC<br>TCCAGATTAAAAAAACAGAATTTCCTGCTGGGGTTGGAGTCGGCTGCTCCCTCCCAAACATCTGTGTACTTCTGTGCCAGCAGTT<br>ACATGGGGCCTGAAGCTTTCTTTGGACAAGGCACCAGACTCACAGTTGTAGAGGACCTGAACAAGGTGTTCCCACCCGAGGTCGC<br>TGTGTTTGAGCCATCAGAAGCAGAGATCTCCCACACCCAAAAGGCCACACTGGTGTGCCTGGCCACAGGCTTCTTCCCCGACCAC<br>GTGGAGCTGAGCTGGTGGGTGAATGGGAAGGAGGTGCACAGTGGGGTCTGCACGGACCCGCAGCCCCTCAAGGAGCAGCCCGCCC<br>TCAATGACTCCAGATACTGCCTGAGCAGCCGCCTGAGGGTCTCGGCCACCTTCTGGCAGAACCCCCGCAACCACTTCCGCTGTCA<br>AGTCCAGTTCTACGGGCTCTCGGAGAATGACGAGTGGACCCAGGATAGGGCCAAACCCGTCACCCAGATCGTCAGCGCCGAGGCC<br>TGGGGTAGAGCAGACTGTGGCTTTACCTCGGTGTCCTACCAGCAAGGGGTCCTGTCTGCCACCATCCTCTATGAGATCCTGCTAG<br>GGAAGGCCACCCTGTATGCTGTGCTGGTCAGCGCCCTTGTGTTGATGGCCATGGTCAAGAGAAAGGATTTCTGA
ND_B-19	ATGGCATGCCCTGGCTTCCTGTGGGCACTTGTGATCTCCACCTGTCTTGAATTTAGCATGGCTCAGACAGTCACT<br>CAGTCTCAACCAGAGATGTCTGTGCAGGAGGCAGAGACCGTGACCCTGAGCTGCACATATGACACCAGTGAGAGT<br>GATTATTATTTATTCTGGTACAAGCAGCCTCCCAGCAGGCAGATGATTCTCGTTATTCGCCAAGAAGCTTATAAG<br>CAACAGAATGCAACAGAGAATCGTTTCTCTGTGAACTTCCAGAAAGCAGCCAAATCCTTCAGTCTCAAGATCTCA<br>GACTCACAGCTGGGGGATGCCGCGATGTATTTCTGTGCTTATAGGAGCGGAAGTGATGGAGGAAGCCAAGGAAAT<br>CTCATCTTTGGAAAAGGCACTAAACTCTCTGTTAAACCAAATATCCAGAACCCTGACCCTGCCGTGTACCAGCTG<br>AGAGACTCTAAATCCAGTGACAAGTCTGTCTGCCTATTCACCGATTTTGATTCTCAAACAAATGTGTCACAAAGT<br>AAGGATTCTGATGTGTATATCACAGACAAATGTGTGCTAGACATGAGGTCTATGGACTTCAAGAGCAACAGTGCT<br>GTGGCCTGGAGCAACAAATCTGACTTTGCATGTGCAAACGCCTTCAACAACAGCATTATTCCAGAAGACACCTTC<br>TTCCCCAGCCCAGAAAGTTCCTGTGATGTCAAGCTGGTCGAGAAAAGCTTTGAAACAGATACGAACCTAAACTTT<br>CAAAACCTGTCAGTGATTGGGTTCCGAATCCTCCTCCTGAAAGTGGCCGGGTTTAATCTGCTCATGACGCTGCGG<br>CTGTGGTCCAGCTGA	ATGGGCTGCAGGCTGCTCTGCTGTGCGGTTCTCTGTCTCCTGGGAGCGGGTGAGTTGGTCCCCATGGAAACGGGAGTTACGCAGA<br>CACCAAGACACCTGGTCATGGGAATGACAAATAAGAAGTCTTTGAAATGTGAACAACATCTGGGTCATAACGCTATGTATTGGTA<br>CAAGCAAAGTGCTAAGAAGCCACTGGAGCTCATGTTTGTCTACAGTCTTGAAGAACGGGTTGAAAACAACAGTGTGCCAAGTCGC<br>TTCTCACCTGAATGCCCCAACAGCTCTCACTTATTCCTTCACCTACACACCCTGCAGCCAGAAGACTCGGCCCTGTATCTCTGCG<br>CCAGCAGCCAAGTTTCGGGTTACGAGCAGTACTTCGGGCCGGGCACCAGGCTCACGGTCACAGAGGACCTGAAAAACGTGTTCCC<br>ACCCGAGGTCGCTGTGTTTGAGCCATCAGAAGCAGAGATCTCCCACACCCAAAAGGCCACACTGGTGTGCCTGGCCACAGGCTTC<br>TACCCCGACCACGTGGAGCTGAGCTGGTGGGTGAATGGGAAGGAGGTGCACAGTGGGGTCTGCACAGACCCGCAGCCCCTCAAGG<br>AGCAGCCCGCCCTCAATGACTCCAGATACTGCCTGAGCAGCCGCCTGAGGGTCTCGGCCACCTTCTGGCAGAACCCCCGCAACCA<br>CTTCCGCTGTCAAGTCCAGTTCTACGGGCTCTCGGAGAATGACGAGTGGACCCAGGATAGGGCCAAACCTGTCACCCAGATCGTC<br>AGCGCCGAGGCCTGGGGTAGAGCAGACTGTGGCTTCACCTCCGAGTCTTACCAGCAAGGGGTCCTGTCTGCCACCATCCTCTATG<br>AGATCTTGCTAGGGAAGGCCACCTTGTATGCCGTGCTGGTCAGTGCCCTCGTGCTGATGGCCATGGTCAAGAGAAAGGATTCCAG<br>AGGC
ND_B-21	ATGTGGGGAGCTTTCCTTCTCTATGTTTCCATGAAGATGGGAGGCACTGCAGGACAAAGCCTTGAGCAGCCCTCT<br>GAAGTGACAGCTGTGGAAGGAGCCATTGTCCAGATAAACTGCACGTACCAGACATCTGGGTTTTATGGGCTGTCC<br>TGGTACCAGCAACATGATGGCGGAGCACCCACATTTCTTTCTTACAATGCTCTGGATGGTTTGGAGGAGACAGGT<br>CGTTTTTCTTCATTCCTTAGTCGCTCTGATAGTTATGGTTACCTCCTTCTACAGGAGCTCCAGATGAAAGACTCT<br>GCCTCTTACTTCTGCGCTGTGAGAGATGACTATGGTCAGAATTTTGTCTTTGGTCCCGGAACCAGATTGTCCGTG<br>CTGCCCTATATCCAGAACCCTGACCCTGCCGTGTACCAGCTGAGAGACTCTAAATCCAGTGACAAGTCTGTCTGC<br>CTATTCACCGATTTTGATTCTCAAACAAATGTGTCACAAAGTAAGGATTCTGATGTGTATATCACAGACAAATGT<br>GTGCTAGACATGAGGTCTATGGACTTCAAGAGCAACAGTGCTGTGGCCTGGAGCAACAAATCTGACTTTGCATGT<br>GCAAACGCCTTCAACAACAGCATTATTCCAGAAGACACCTTCTTCCCCAGCCCAGAAAGTTCCTGTGATGTCAAG<br>CTGGTCGAGAAAAGCTTTGAAACAGATACGAACCTAAACTTTCAAAACCTGTCAGTGATTGGGTTCCGAATCCTC<br>CTCCTGAAAGTGGCCGGGTTTAATCTGCTCATGACGCTGCGGCTGTGGTCCAGCTGA	ATGGACACCAGAGTACTCTGCTGTGCGGTCATCTGTCTTCTGGGGGCAGGTCTCTCAAATGCCGGCGTCATGCAGAACCCAAGAC<br>ACCTGGTCAGGAGGAGGGGACAGGAGGCAAGACTGAGATGCAGCCCAATGAAAGGACACAGTCATGTTTACTGGTATCGGCAGCT<br>CCCAGAGGAAGGTCTGAAATTCATGGTTTATCTCCAGAAAGAAAATATCATAGATGAGTCAGGAATGCCAAAGGAACGATTTTCT<br>GCTGAATTTCCCAAAGAGGGCCCCAGCATCCTGAGGATCCAGCAGGTAGTGCGAGGAGATTCGGCAGCTTATTTCTGTGCCAGCT<br>CACCACAGGGTGACAATGAGCAGTTCTTCGGGCCAGGGACACGGCTCACCGTGCTAGAGGACCTGAACAAGGTGTTCCCACCCGA<br>GGTCGCTGTGTTTGAGCCATCAGAAGCAGAGATCTCCCACACCCAAAAGGCCACACTGGTGTGCCTGGCCACAGGCTTCTTCCCC<br>GACCACGTGGAGCTGAGCTGGTGGGTGAATGGGAAGGAGGTGCACAGTGGGGTCTGCACGGACCCGCAGCCCCTCAAGGAGCAGC<br>CCGCCCTCAATGACTCCAGATACTGCCTGAGCAGCCGCCTGAGGGTCTCGGCCACCTTCTGGCAGAACCCCCGCAACCACTTCCG<br>CTGTCAAGTCCAGTTCTACGGGCTCTCGGAGAATGACGAGTGGACCCAGGATAGGGCCAAACCCGTCACCCAGATCGTCAGCGCC<br>GAGGCCTGGGGTAGAGCAGACTGTGGCTTTACCTCGGTGTCCTACCAGCAAGGGGTCCTGTCTGCCACCATCCTCTATGAGATCC<br>TGCTAGGGAAGGCCACCCTGTATGCTGTGCTGGTCAGCGCCCTTGTGTTGATGGCCATGGTCAAGAGAAAGGATTTCTGA

Supplementary Table 5. Codon-optimized nucleotide sequences of 5T4-specific TCRs

Clone	TRA	TRB
ND_A-2	ATGAAGTCCCTGCGGGTGCTGCTCGTGATCCTGTGGCTCCAGCTGAGCTGGGTGTGGTCCCAGCAGAAAGA<br>GGTGGAACAGAACAGCGGCCCTCTGAGCGTGCCAGAAGGCGCTATCGCCAGCCTGAACTGCACCTACAGCG<br>ACAGAGGCAGCCAGAGCTTCTTCTGGTACAGACAGTACAGCGGCAAGAGCCCCGAGCTGATCATGTTCATC<br>TACAGCAACGGCGACAAAGAGGACGGCCGGTTCACCGCCCAGCTGAACAAGGCCAGCCAGTACGTGTCCCT<br>GCTGATCAGAGACAGCCAGCCCAGCGACAGCGCCACCTATCTGTGTGCCGTGCCTGACGACGCCGGCAACA<br>TGCTGACATTTGGCGGCGGAACCCGGCTGATGGTCAAGCCCCGATATCCAGAACCCTGACCCTGCCGTGTA<br>CCAGCTGAGAGACTCTAAATCCAGTGACAAGTCTGTCTGCCTATTCACCGATTTTGATTCTCAAACAAATG<br>TGTCACAAAGTAAGGATTCTGATGTGTATATCACAGACAAATGTGTGCTAGACATGAGGTCTATGGACTTC<br>AAGAGCAACAGTGCTGTGGCCTGGAGCAACAAATCTGACTTTGCATGTGCAAACGCCTTCAACAACAGCAT<br>TATTCCAGAAGACACCTTCTTCCCCAGCCCAGAAAGTTCCTGTGATGTCAAGCTGGTCGAGAAAAGCTTTG<br>AAACAGATACGAACCTAAACTTTCAAAACCTGTCAGTGATTGGGTTCCGAATCCTCCTCCTGAAAGTGGCC<br>GGGTTTAATCTGCTCATGACGCTGCGGCTGTGGTCCAGCTGA	ATGGGCACCAGACTGTTTTTCTACGTGGCCCTGTGCCTGCTGTGGGCCGGACATAGAGATGCCGGAATCACCCAGAGCCCCCGGT<br>ACAAGATCACCGAGACAGGCAGACAAGTGACCCTGATGTGCCACCAGACCTGGTCCCACAGCTACATGTTCTGGTACAGACAGGA<br>CCTGGGCCACGGCCTGCGGCTGATCTACTATTCTGCCGCCGCTGACATCACCGACAAGGGCGAAGTGCCCGACGGCTACGTGGTG<br>TCCAGAAGCAAGACCGAGAACTTCCCACTGACCCTGGAAAGCGCCACCCGGTCCCAGACCAGCGTGTACTTTTGTGCCAGCAGCG<br>AGCTGCCCGCTGGCGGCACCAATGAGCAGTTTTTTGGCCCTGGCACCCGGCTGACCGTGCTGGACCTGAAGAATGTGTTCCCACC<br>CGAGGTCGCTGTGTTTGAGCCATCAGAAGCAGAGATCTCCCACACCCAAAAGGCCACACTGGTGTGCCTGGCCACAGGCTTCTTC<br>CCCGACCACGTGGAGCTGAGCTGGTGGGTGAATGGGAAGGAGGTGCACAGTGGGGTCTGCACGGACCCGCAGCCCCTCAAGGAGC<br>AGCCCGCCCTCAATGACTCCAGATACTGCCTGAGCAGCCGCCTGAGGGTCTCGGCCACCTTCTGGCAGAACCCCCGCAACCACTT<br>CCGCTGTCAAGTCCAGTTCTACGGGCTCTCGGAGAATGACGAGTGGACCCAGGATAGGGCCAAACCCGTCACCCAGATCGTCAGC<br>GCCGAGGCCTGGGGTAGAGCAGACTGTGGCTTTACCTCGGTGTCCTACCAGCAAGGGGTCCTGTCTGCCACCATCCTCTATGAGA<br>TCCTGCTAGGGAAGGCCACCCTGTATGCTGTGCTGGTCAGCGCCCTTGTGTTGATGGCCATGGTCAAGAGAAAGGATTTCTGA
ND_C-3	ATGGCCTGCCCCGGATTTCTGTGGGCCCTCGTGATCAGCACCTGTCTGGAATTCAGCATGGCCCAGACCGT<br>GACCCAGAGCCAGCCTGAGATGAGCGTGCAGGAAGCCGAGACAGTGACCCTGAGCTGCACCTACGACACCA<br>GCGAGAGCGACTACTACCTGTTCTGGTACAAGCAGCCCCCCAGCCGGCAGATGATCCTCGTGATTAGACAG<br>GAAGCCTATAAGCAGCAGAACGCCACCGAGAACAGATTCAGCGTGAACTTCCAGAAGGCCGCCAAGAGCTT<br>CAGCCTGAAGATCAGCGACAGCCAGCTGGGCGACGCCGCCATGTACTTTTGTTCTAGCGGCGGAGGCGCCG<br>ACGGCCTGACATTTGGAAAGGGCACCCACCTGATCATCCAGCCCTGATATCCAGAACCCTGACCCTGCCGT<br>GTACCAGCTGAGAGACTCTAAATCCAGTGACAAGTCTGTCTGCCTATTCACCGATTTTGATTCTCAAACAA<br>ATGTGTCACAAAGTAAGGATTCTGATGTGTATATCACAGACAAATGTGTGCTAGACATGAGGTCTATGGAC<br>TTCAAGAGCAACAGTGCTGTGGCCTGGAGCAACAAATCTGACTTTGCATGTGCAAACGCCTTCAACAACAG<br>CATTATTCCAGAAGACACCTTCTTCCCCAGCCCAGAAAGTTCCTGTGATGTCAAGCTGGTCGAGAAAAGCT<br>TTGAAACAGATACGAACCTAAACTTTCAAAACCTGTCAGTGATTGGGTTCCGAATCCTCCTCCTGAAAGTG<br>GCCGGGTTTAATCTGCTCATGACGCTGCGGCTGTGGTCCAGCTGA	ATGTCTCTGGGACTGCTGTGTTGCGGCGCCTTCAGCCTGCTGTGGGCCGGACCTGTGAATGCCGGCGTGACCCAGACCCCCAAGT<br>TCCGGGTGCTGAAAACCGGCCAGAGCATGACCCTGCTGTGCGCCCAGGACATGAACCACGAGTACATGTATTGGTACAGACAGGA<br>CCCCGGCATGGGCCTGCGGCTGATCCACTATTCTGTGGGCGAGGGCACCACCGCCAAGGGCGAAGTGCCTGATGGCTACAACGTG<br>TCCCGGCTGAAGAAGCAGAACTTCCTGCTGGGCCTGGAAAGCGCCGCTCCTAGCCAGACCAGCGTGTACTTCTGCGCCAGCATGG<br>ACCTGGCCTTCAAGCAGTACTTCGGCCCTGGCACCAGACTGACCGTGACCGAGGACCTGAAGAACGTGTTCCCACCCGAGGTCGC<br>TGTGTTTGAGCCATCAGAAGCAGAGATCTCCCACACCCAAAAGGCCACACTGGTGTGCCTGGCCACAGGCTTCTTCCCCGACCAC<br>GTGGAGCTGAGCTGGTGGGTGAATGGGAAGGAGGTGCACAGTGGGGTCTGCACGGACCCGCAGCCCCTCAAGGAGCAGCCCGCCC<br>TCAATGACTCCAGATACTGCCTGAGCAGCCGCCTGAGGGTCTCGGCCACCTTCTGGCAGAACCCCCGCAACCACTTCCGCTGTCA<br>AGTCCAGTTCTACGGGCTCTCGGAGAATGACGAGTGGACCCAGGATAGGGCCAAACCCGTCACCCAGATCGTCAGCGCCGAGGCC<br>TGGGGTAGAGCAGACTGTGGCTTTACCTCGGTGTCCTACCAGCAAGGGGTCCTGTCTGCCACCATCCTCTATGAGATCCTGCTAG<br>GGAAGGCCACCCTGTATGCTGTGCTGGTCAGCGCCCTTGTGTTGATGGCCATGGTCAAGAGAAAGGATTTCTGA
KCD_D-6	ATGGCCTGCCCCGGATTTCTGTGGGCCCTCGTGATCAGCACCTGTCTGGAATTCAGCATGGCCCAGACCGT<br>GACCCAGAGCCAGCCTGAGATGAGCGTGCAGGAAGCCGAGACAGTGACCCTGAGCTGCACCTACGACACCA<br>GCGAGAGCGACTACTACCTGTTCTGGTACAAGCAGCCCCCCAGCCGGCAGATGATCCTCGTGATTAGACAG<br>GAAGCCTATAAGCAGCAGAACGCCACCGAGAACAGATTCAGCGTGAACTTCCAGAAGGCCGCCAAGAGCTT<br>CAGCCTGAAGATCAGCGACAGCCAGCTGGGCGACGCCGCCATGTACTTTTGTGCTAGCGGAGGCGGAGCCG<br>ACGGCCTGACATTTGGCAAGGGCACCCACCTGATCATCCAGCCCTATATCCAGAACCCTGACCCTGCCGTG<br>TACCAGCTGAGAGACTCTAAATCCAGTGACAAGTCTGTCTGCCTATTCACCGATTTTGATTCTCAAACAAA<br>TGTGTCACAAAGTAAGGATTCTGATGTGTATATCACAGACAAATGTGTGCTAGACATGAGGTCTATGGACT<br>TCAAGAGCAACAGTGCTGTGGCCTGGAGCAACAAATCTGACTTTGCATGTGCAAACGCCTTCAACAACAGC<br>ATTATTCCAGAAGACACCTTCTTCCCCAGCCCAGAAAGTTCCTGTGATGTCAAGCTGGTCGAGAAAAGCTT<br>TGAAACAGATACGAACCTAAACTTTCAAAACCTGTCAGTGATTGGGTTCCGAATCCTCCTCCTGAAAGTGG<br>CCGGGTTTAATCTGCTCATGACGCTGCGGCTGTGGTCCAGCTGA	ATGTCTCTGGGACTGCTGTGTTGCGGCGCCTTCAGCCTGCTGTGGGCCGGACCTGTGAATGCCGGCGTGACCCAGACCCCCAAGT<br>TCCGGGTGCTGAAAACCGGCCAGAGCATGACCCTGCTGTGCGCCCAGGACATGAACCACGAGTACATGTATTGGTACAGACAGGA<br>CCCCGGCATGGGCCTGCGGCTGATCCACTATTCTGTGGGCGAGGGCACCACCGCCAAGGGCGAAGTGCCTGATGGCTACAACGTG<br>TCCCGGCTGAAGAAGCAGAACTTCCTGCTGGGCCTGGAAAGCGCCGCTCCTAGCCAGACCAGCGTGTACTTCTGCGCCAGCAGCT<br>TCCTGACCGACACCCAGTACTTTGGCCCTGGCACCAGACTGACCGTGCTGGAAGATCTGAAGAACGTGTTCCCACCCGAGGTCGC<br>TGTGTTTGAGCCATCAGAAGCAGAGATCTCCCACACCCAAAAGGCCACACTGGTGTGCCTGGCCACAGGCTTCTTCCCCGACCAC<br>GTGGAGCTGAGCTGGTGGGTGAATGGGAAGGAGGTGCACAGTGGGGTCTGCACGGACCCGCAGCCCCTCAAGGAGCAGCCCGCCC<br>TCAATGACTCCAGATACTGCCTGAGCAGCCGCCTGAGGGTCTCGGCCACCTTCTGGCAGAACCCCCGCAACCACTTCCGCTGTCA<br>AGTCCAGTTCTACGGGCTCTCGGAGAATGACGAGTGGACCCAGGATAGGGCCAAACCCGTCACCCAGATCGTCAGCGCCGAGGCC<br>TGGGGTAGAGCAGACTGTGGCTTTACCTCGGTGTCCTACCAGCAAGGGGTCCTGTCTGCCACCATCCTCTATGAGATCCTGCTAG<br>GGAAGGCCACCCTGTATGCTGTGCTGGTCAGCGCCCTTGTGTTGATGGCCATGGTCAAGAGAAAGGATTTCTGA
ND_A-15	ATGACACGAGTTAGCTTGCTGTGGGCAGTCGTGGTCTCCACCTGTCTTGAATCCGGCATGGCCCAGACAGT<br>CACTCAGTCTCAACCAGAGATGTCTGTGCAGGAGGCAGAGACTGTGACCCTGAGTTGCACATATGACACCA<br>GTGAGAATAATTATTATTTGTTCTGGTACAAGCAGCCTCCCAGCAGGCAGATGATTCTCGTTATTCGCCAA<br>GAAGCTTATAAGCAACAGAATGCAACGGAGAATCGTTTCTCTGTGAACTTCCAGAAAGCAGCCAAATCCTT<br>CAGTCTCAAGATCTCAGACTCACAGCTGGGGGACACTGCGATGTATTTCTGTGCCTCGATGTATTCAGGAG<br>GAGGTGCTGACGGACTCACCTTTGGCAAAGGGACTCATCTAATCATCCAGCCCTGATATCCAGAACCCTGA<br>CCCTGCCGTGTACCAGCTGAGAGACTCTAAATCCAGTGACAAGTCTGTCTGCCTATTCACCGATTTTGATT<br>CTCAAACAAATGTGTCACAAAGTAAGGATTCTGATGTGTATATCACAGACAAATGTGTGCTAGACATGAGG<br>TCTATGGACTTCAAGAGCAACAGTGCTGTGGCCTGGAGCAACAAATCTGACTTTGCATGTGCAAACGCCTT<br>CAACAACAGCATTATTCCAGAAGACACCTTCTTCCCCAGCCCAGAAAGTTCCTGTGATGTCAAGCTGGTCG<br>AGAAAAGCTTTGAAACAGATACGAACCTAAACTTTCAAAACCTGTCAGTGATTGGGTTCCGAATCCTCCTC<br>CTGAAAGTGGCCGGGTTTAATCTGCTCATGACGCTGCGGCTGTGGTCCAGCTGA	ATGGGACCTCAGCTGCTGGGATACGTGGTGCTGTGTCTGCTGGGAGCCGGACCTCTGGAAGCCCAAGTGACCCAGAACCCCAGAT<br>ACCTGATCACCGTGACCGGCAAGAAACTGACCGTGACCTGCTCCCAGAACATGAACCACGAGTACATGAGCTGGTACAGACAGGA<br>CCCCGGCCTGGGCCTGCGGCAGATCTACTACAGCATGAACGTGGAAGTGACCGACAAGGGCGACGTGCCCGAGGGCTACAAGGTG<br>TCCCGGAAAGAGAAGCGGAACTTCCCACTGATCCTGGAAAGCCCCAGCCCCAACCAGACCAGCCTGTACTTCTGCGCCAGCAGCT<br>TCTTCAGCAACACCGGCGAGCTGTTCTTCGGCGAGGGCAGCAGACTGACAGTGCTGGAAGATCTGAACAAGGTGTTCCCACCCGA<br>GGTCGCTGTGTTTGAGCCATCAGAAGCAGAGATCTCCCACACCCAAAAGGCCACACTGGTGTGCCTGGCCACAGGCTTCTTCCCC<br>GACCACGTGGAGCTGAGCTGGTGGGTGAATGGGAAGGAGGTGCACAGTGGGGTCTGCACGGACCCGCAGCCCCTCAAGGAGCAGC<br>CCGCCCTCAATGACTCCAGATACTGCCTGAGCAGCCGCCTGAGGGTCTCGGCCACCTTCTGGCAGAACCCCCGCAACCACTTCCG<br>CTGTCAAGTCCAGTTCTACGGGCTCTCGGAGAATGACGAGTGGACCCAGGATAGGGCCAAACCCGTCACCCAGATCGTCAGCGCC<br>GAGGCCTGGGGTAGAGCAGACTGTGGCTTTACCTCGGTGTCCTACCAGCAAGGGGTCCTGTCTGCCACCATCCTCTATGAGATCC<br>TGCTAGGGAAGGCCACCCTGTATGCTGTGCTGGTCAGCGCCCTTGTGTTGATGGCCATGGTCAAGAGAAAGGATTTCTGA
ND_C-17	ATGGCCTGCCCCGGATTTCTGTGGGCCCTCGTGATCAGCACCTGTCTGGAATTCAGCATGGCCCAGACCGT<br>GACCCAGAGCCAGCCTGAGATGAGCGTGCAGGAAGCCGAGACAGTGACCCTGAGCTGCACCTACGACACCA<br>GCGAGAGCGACTACTACCTGTTCTGGTACAAGCAGCCCCCCAGCCGGCAGATGATCCTCGTGATTAGACAG<br>GAAGCCTATAAGCAGCAGAACGCCACCGAGAACAGATTCAGCGTGAACTTCCAGAAGGCCGCCAAGAGCTT<br>CAGCCTGAAGATCAGCGACAGCCAGCTGGGCGACGCCGCCATGTATTTTTGTGCTGGCGGAGGCGGAGCCG<br>ACGGCCTGACATTTGGAAAGGGCACCCACCTGATCATCCAGCCCTATATCCAGAACCCTGACCCTGCCGTG<br>TACCAGCTGAGAGACTCTAAATCCAGTGACAAGTCTGTCTGCCTATTCACCGATTTTGATTCTCAAACAAA<br>TGTGTCACAAAGTAAGGATTCTGATGTGTATATCACAGACAAATGTGTGCTAGACATGAGGTCTATGGACT<br>TCAAGAGCAACAGTGCTGTGGCCTGGAGCAACAAATCTGACTTTGCATGTGCAAACGCCTTCAACAACAGC<br>ATTATTCCAGAAGACACCTTCTTCCCCAGCCCAGAAAGTTCCTGTGATGTCAAGCTGGTCGAGAAAAGCTT<br>TGAAACAGATACGAACCTAAACTTTCAAAACCTGTCAGTGATTGGGTTCCGAATCCTCCTCCTGAAAGTGG<br>CCGGGTTTAATCTGCTCATGACGCTGCGGCTGTGGTCCAGCTGA	ATGTCTCTGGGACTGCTGTGTTGCGGCGCCTTCAGCCTGCTGTGGGCCGGACCTGTGAATGCCGGCGTGACCCAGACCCCCAAGT<br>TCCGGGTGCTGAAAACCGGCCAGAGCATGACCCTGCTGTGCGCCCAGGACATGAACCACGAGTACATGTATTGGTACAGACAGGA<br>CCCCGGCATGGGCCTGCGGCTGATCCACTATTCTGTGGGCGAGGGCACCACCGCCAAGGGCGAAGTGCCTGATGGCTACAACGTG<br>TCCCGGCTGAAGAAGCAGAACTTCCTGCTGGGCCTGGAAAGCGCCGCTCCTAGCCAGACCAGCGTGTACTTCTGCGCCAGCAGCT<br>ATATGGGCCCCGAGGCATTCTTTGGGCAGGGAACCAGACTGACCGTGGTGGAAGATCTGAACAAGGTGTTCCCACCCGAGGTCGC<br>TGTGTTTGAGCCATCAGAAGCAGAGATCTCCCACACCCAAAAGGCCACACTGGTGTGCCTGGCCACAGGCTTCTTCCCCGACCAC<br>GTGGAGCTGAGCTGGTGGGTGAATGGGAAGGAGGTGCACAGTGGGGTCTGCACGGACCCGCAGCCCCTCAAGGAGCAGCCCGCCC<br>TCAATGACTCCAGATACTGCCTGAGCAGCCGCCTGAGGGTCTCGGCCACCTTCTGGCAGAACCCCCGCAACCACTTCCGCTGTCA<br>AGTCCAGTTCTACGGGCTCTCGGAGAATGACGAGTGGACCCAGGATAGGGCCAAACCCGTCACCCAGATCGTCAGCGCCGAGGCC<br>TGGGGTAGAGCAGACTGTGGCTTTACCTCGGTGTCCTACCAGCAAGGGGTCCTGTCTGCCACCATCCTCTATGAGATCCTGCTAG<br>GGAAGGCCACCCTGTATGCTGTGCTGGTCAGCGCCCTTGTGTTGATGGCCATGGTCAAGAGAAAGGATTTCTGA
ND_B-19	ATGGCCTGCCCCGGATTTCTGTGGGCCCTCGTGATCAGCACCTGTCTGGAATTCAGCATGGCCCAGACCGT<br>GACCCAGAGCCAGCCTGAGATGAGCGTGCAGGAAGCCGAGACAGTGACCCTGAGCTGCACCTACGACACCA<br>GCGAGAGCGACTACTACCTGTTCTGGTACAAGCAGCCCCCCAGCCGGCAGATGATCCTCGTGATTAGACAG<br>GAAGCCTATAAGCAGCAGAACGCCACCGAGAACAGATTCAGCGTGAACTTCCAGAAGGCCGCCAAGAGCTT<br>CAGCCTGAAGATCAGCGACAGCCAGCTGGGCGACGCCGCCATGTACTTTTGCGCCTATAGAAGCGGCAGCG<br>ACGGCGGCTCTCAGGGCAATCTGATCTTCGGCAAGGGCACCAAGCTGAGCGTGAAGCCCAGATATCCAGAA<br>CCCTGACCCTGCCGTGTACCAGCTGAGAGACTCTAAATCCAGTGACAAGTCTGTCTGCCTATTCACCGATT<br>TTGATTCTCAAACAAATGTGTCACAAAGTAAGGATTCTGATGTGTATATCACAGACAAATGTGTGCTAGAC<br>ATGAGGTCTATGGACTTCAAGAGCAACAGTGCTGTGGCCTGGAGCAACAAATCTGACTTTGCATGTGCAAA<br>CGCCTTCAACAACAGCATTATTCCAGAAGACACCTTCTTCCCCAGCCCAGAAAGTTCCTGTGATGTCAAGC<br>TGGTCGAGAAAAGCTTTGAAACAGATACGAACCTAAACTTTCAAAACCTGTCAGTGATTGGGTTCCGAATC<br>CTCCTCCTGAAAGTGGCCGGGTTTAATCTGCTCATGACGCTGCGGCTGTGGTCCAGCTGA	ATGGGATGTAGACTGCTGTGTTGCGCCGTGCTGTGCCTGCTGGGAGCTGGCGAACTGGTGCCTATGGAAACCGGCGTGACCCAGA<br>CCCCCAGACACCTCGTGATGGGCATGACCAACAAGAAAAGCCTGAAGTGCGAGCAGCACCTGGGCCACAACGCCATGTACTGGTA<br>CAAGCAGAGCGCCAAGAAACCCCTGGAACTGATGTTCGTGTACAGCCTGGAAGAGAGGGTGGAAAACAACAGCGTGCCCAGCCGG<br>TTCAGCCCCGAGTGCCCTAATAGCAGCCACCTGTTTCTGCATCTGCACACCCTCCAGCCCGAGGACAGCGCCCTGTATCTGTGTG<br>CCAGCTCTCAGGTGTCCGGCTACGAGCAGTACTTCGGCCCTGGCACCAGACTGACCGTGACCGAGGACCTGAAGAACGTGTTCCC<br>ACCCGAGGTCGCTGTGTTTGAGCCATCAGAAGCAGAGATCTCCCACACCCAAAAGGCCACACTGGTGTGCCTGGCCACAGGCTTC<br>TTCCCCGACCACGTGGAGCTGAGCTGGTGGGTGAATGGGAAGGAGGTGCACAGTGGGGTCTGCACGGACCCGCAGCCCCTCAAGG<br>AGCAGCCCGCCCTCAATGACTCCAGATACTGCCTGAGCAGCCGCCTGAGGGTCTCGGCCACCTTCTGGCAGAACCCCCGCAACCA<br>CTTCCGCTGTCAAGTCCAGTTCTACGGGCTCTCGGAGAATGACGAGTGGACCCAGGATAGGGCCAAACCCGTCACCCAGATCGTC<br>AGCGCCGAGGCCTGGGGTAGAGCAGACTGTGGCTTTACCTCGGTGTCCTACCAGCAAGGGGTCCTGTCTGCCACCATCCTCTATG<br>AGATCCTGCTAGGGAAGGCCACCCTGTATGCTGTGCTGGTCAGCGCCCTTGTGTTGATGGCCATGGTCAAGAGAAAGGATTTCTG<br>A
ND_B-21	ATGTGGGGCGCCTTTCTGCTGTACGTGTCCATGAAGATGGGCGGCACAGCCGGCCAGAGCCTGGAACAGCC<br>TTCTGAAGTGACCGCCGTGGAAGGCGCCATCGTGCAGATCAACTGCACCTACCAGACCAGCGGCTTCTACG<br>GCCTGAGCTGGTATCAGCAGCATGACGGCGGAGCCCCCACCTTCCTGAGCTACAATGCCCTGGACGGCCTG<br>GAAGAGACAGGCCGGTTCAGCAGCTTCCTGTCCAGAAGCGACAGCTACGGCTACCTGCTGCTCCAGGAACT<br>CCAGATGAAGGACAGCGCCAGCTACTTCTGCGCCGTGCGGGATGACTACGGCCAGAACTTCGTGTTCGGCC<br>CTGGCACCAGACTGAGCGTGCTGCCCTATATCCAGAACCCTGACCCTGCCGTGTACCAGCTGAGAGACTCT<br>AAATCCAGTGACAAGTCTGTCTGCCTATTCACCGATTTTGATTCTCAAACAAATGTGTCACAAAGTAAGGA<br>TTCTGATGTGTATATCACAGACAAATGTGTGCTAGACATGAGGTCTATGGACTTCAAGAGCAACAGTGCTG<br>TGGCCTGGAGCAACAAATCTGACTTTGCATGTGCAAACGCCTTCAACAACAGCATTATTCCAGAAGACACC<br>TTCTTCCCCAGCCCAGAAAGTTCCTGTGATGTCAAGCTGGTCGAGAAAAGCTTTGAAACAGATACGAACCT<br>AAACTTTCAAAACCTGTCAGTGATTGGGTTCCGAATCCTCCTCCTGAAAGTGGCCGGGTTTAATCTGCTCA<br>TGACGCTGCGGCTGTGGTCCAGCTGA	ATGGATACAAGAGTGCTGTGCTGCGCCGTGATCTGCCTGCTGGGAGCCGGACTGTCTAATGCCGGCGTGATGCAGAACCCCAGAC<br>ACCTCGTGCGGCGGAGAGGACAGGAAGCCAGACTGCGCTGTAGCCCCATGAAGGGCCACAGCCACGTGTACTGGTACAGACAGCT<br>GCCCGAAGAGGGCCTGAAGTTCATGGTGTACCTCCAGAAAGAGAACATCATCGACGAGAGCGGCATGCCCAAAGAGCGGTTCAGC<br>GCCGAGTTCCCCAAAGAGGGCCCCAGCATCCTGAGAATCCAGCAGGTCGTGCGGGGCGATAGCGCCGCCTATTTTTGTGCCAGCA<br>GCCCCCAGGGCGACAACGAGCAGTTTTTTGGCCCTGGCACCCGGCTGACCGTGCTGGAAGATCTGAACAAGGTGTTCCCACCCGA<br>GGTCGCTGTGTTTGAGCCATCAGAAGCAGAGATCTCCCACACCCAAAAGGCCACACTGGTGTGCCTGGCCACAGGCTTCTTCCCC<br>GACCACGTGGAGCTGAGCTGGTGGGTGAATGGGAAGGAGGTGCACAGTGGGGTCTGCACGGACCCGCAGCCCCTCAAGGAGCAGC<br>CCGCCCTCAATGACTCCAGATACTGCCTGAGCAGCCGCCTGAGGGTCTCGGCCACCTTCTGGCAGAACCCCCGCAACCACTTCCG<br>CTGTCAAGTCCAGTTCTACGGGCTCTCGGAGAATGACGAGTGGACCCAGGATAGGGCCAAACCCGTCACCCAGATCGTCAGCGCC<br>GAGGCCTGGGGTAGAGCAGACTGTGGCTTTACCTCGGTGTCCTACCAGCAAGGGGTCCTGTCTGCCACCATCCTCTATGAGATCC<br>TGCTAGGGAAGGCCACCCTGTATGCTGTGCTGGTCAGCGCCCTTGTGTTGATGGCCATGGTCAAGAGAAAGGATTTCTGA
